# Production of diverse brGDGTs by Acidobacterium *Solibacter usitatus* in response to temperature, pH, and O_2_ provides a culturing perspective on brGDGT proxies and biosynthesis

**DOI:** 10.1111/gbi.12525

**Published:** 2022-09-23

**Authors:** Toby A. Halamka, Jonathan H. Raberg, Jamie M. McFarlin, Adam D. Younkin, Christopher Mulligan, Xiao‐Lei Liu, Sebastian H. Kopf

**Affiliations:** ^1^ Department of Geological Sciences University of Colorado Boulder Denver CO USA; ^2^ Faculty of Earth Sciences University of Iceland Reykjavik Iceland; ^3^ School of Geosciences University of Oklahoma Norman Oklahoma USA

**Keywords:** Acidobacteria, brGDGTs, paleoclimate

## Abstract

Branched glycerol dialkyl glycerol tetraethers (brGDGTs) are bacterial membrane lipids that are frequently employed as paleoenvironmental proxies because of the strong empirical correlations between their relative abundances and environmental temperature and pH. Despite the ubiquity of brGDGTs in modern and paleoenvironments, the source organisms of these enigmatic compounds have remained elusive, requiring paleoenvironmental applications to rely solely on observed environmental correlations. Previous laboratory and environmental studies have suggested that the globally abundant bacterial phylum of the Acidobacteria may be an important brGDGT producer in nature. Here, we report on experiments with a cultured Acidobacterium, *Solibacter usitatus*, that makes a large portion of its cellular membrane (24 ± 9% across all experiments) out of a structurally diverse set of tetraethers including the common brGDGTs Ia, IIa, IIIa, Ib, and IIb. *Solibacter usitatus* was grown across a range of conditions including temperatures from 15 to 30°C, pH from 5.0 to 6.5, and O_2_ from 1% to 21%, and demonstrated pronounced shifts in the degree of brGDGT methylation across these growth conditions. The temperature response in culture was in close agreement with trends observed in published environmental datasets, supporting a physiological basis for the empirical relationship between brGDGT methylation number and temperature. However, brGDGT methylation at lower temperatures (15 and 20°C) was modulated by culture pH with higher pH systematically increasing the degree of methylation. In contrast, pH had little effect on brGDGT cyclization, supporting the hypothesis that changes in bacterial community composition may underlie the link between cyclization number and pH observed in environmental samples. Oxygen concentration likewise affected brGDGT methylation highlighting the potential for this environmental parameter to impact paleotemperature reconstruction. Low O_2_ culture conditions further resulted in the production of uncommon brGDGT isomers that could be indicators of O_2_ limitation. Finally, the production of brGTGTs (trialkyl tetraethers) in addition to the previously discovered iso‐C15‐based mono‐ and diethers in *S. usitatus* suggests a potential biosynthetic pathway for brGDGTs that uses homologs of the archaeal tetraether synthase (Tes) enzyme for tetraether synthesis from diethers.

## 1 INTRODUCTION

Branched glycerol dialkyl glycerol tetraethers (brGDGTs) are a group of membrane‐spanning non‐isoprenoidal lipid biomarkers first characterized from peat (Sinninghe Damsté et al., 2000) and since discovered in virtually all modern environments including soils, lakes, rivers, hydrothermal settings, marine environments, and sedimentary systems (De Jonge, Stadnitskaia, et al., 2014; Hopmans et al., 2004; Lincoln et al., 2013; Raberg et al., 2022a; Tierney & Russell, 2009; Weijers et al., 2006). Today, several structural variations of brGDGTs that differ in the number of cyclopentyl moieties, the number of methyl branches, and the position of some of the branches are routinely quantified in environmental samples and frequently used for paleoenvironmental reconstruction (e.g., Lauretano et al., 2021; Lindberg et al., 2022; Lu et al., 2019; Naafs, Gallego‐Sala, et al., 2017; Peterse et al., 2012; Weijers et al., 2007).

In environmental brGDGTs, the number of alkyl‐chain methylations correlates strongly with temperature in numerous sample types, including soils (e.g., Naafs, Inglis, et al., 2017), peats (e.g., Naafs, Gallego‐Sala, et al., 2017), lake sediments (e.g., Martínez‐Sosa et al., 2021; Raberg et al., 2021), and marine sediments (Xiao et al., 2022). These changes in the number of methylations are commonly quantified by calculating indices such as the methylation index of branched tetraethers (MBT; Weijers et al., 2007; and MBT′_5Me_ De Jonge, Hopmans, et al., 2014) or by grouping brGDGTs into the structurally based methylation (Meth) set (Raberg et al., 2021) for comparisons with environmental temperatures. Similarly, a correlation has been observed between pH and brGDGT cyclopentane ring number, as demonstrated by the cyclization index of branched tetraethers (CBT) and related indices (e.g., CBT_5Me_ and CBT′; De Jonge, Hopmans, et al., 2014) or the cyclization (Cyc) set (Raberg et al., 2021). Additionally, both pH (e.g., through the isomer ratio index; Dang et al., 2016; De Jonge, Hopmans, et al., 2014) and salinity/conductivity (e.g., Raberg et al., 2021; Wang et al., 2021) have been shown to correlate with the positions of alkyl‐chain methylations. Finally, other environmental parameters, most notably dissolved oxygen (Liu et al., 2014; Martínez‐Sosa & Tierney, 2019; Weber et al., 2018; Wu et al., 2021; Yao et al., 2020), can influence brGDGT distributions in nature, adding complexity to the observed relationships between structural distributions and temperature/pH, posing both new challenges and new opportunities for proxy applications.

Despite more than 20 years of work on environmental brGDGTs, the source organisms of these ubiquitous compounds remain largely unknown. Though brGDGTs are structurally similar to membrane‐spanning isoprenoidal glycerol tetraethers produced by Archaea, the stereochemistry of the glycerol backbone of brGDGTs points to a bacterial source (Weijers et al., 2006). Among the myriad bacterial heterotrophs that exist in nature, the phylum Acidobacteria has gained the most attention as a potential source group of environmental brGDGTs. In soil environments, Acidobacteria frequently represent more than 20% of all classified bacterial sequences and as high as 70% in some acidic environments (Jones et al., 2009), with community sequencing in environmental samples and laboratory mesocosms showing strong correlations between Acidobacteria populations and the production of brGDGTs (De Jonge et al., 2021; Martínez‐Sosa & Tierney, 2019; Weijers et al., 2010).

Unfortunately, the isolation and subsequent laboratory cultivation of Acidobacteria have proven difficult, resulting in only a small pool of cultured representatives (George et al., 2011) with still no pure cultures available for more than half of the 26 major taxonomic subdivisions (SDs; Barns et al., 2007). Insights about the physiology and likely heterotrophic, oligotrophic, and mostly aerobic lifestyle of the Acidobacteria are thus largely built on genomic analyses (Eichorst et al., 2018) and culturing work with a relatively small group of SD 1, 3, 4, 6, and 8 pure cultures. Laboratory studies with the available strains revealed several likely brGDGT precursor lipids found within the phylum (Sinninghe Damsté et al., 2011) including abundant ether‐bound lipids in SD 4 cultures (Sinninghe Damsté et al., 2014, 2018), and the identification of at least one common brGDGT (brGDGT Ia) in two SD 1 strains (Sinninghe Damsté et al., 2011) that is synthesized in response to low O_2_ in one of the two strains (Halamka et al., 2021).

Despite these discoveries and ongoing efforts to isolate new Acidobacteria and other soil microorganisms, no organisms that produce the entire range of brGDGT structures found in nature and used in proxy calibrations have emerged. Other phyla of soil bacteria, often with equally poor representation in culture collections as Acidobacteria, cannot be dismissed as potential brGDGT producers. Acidobacteria are not nearly as abundant in the bacterial communities of some other environments, including lakes (van Bree et al., 2020; Weber et al., 2018), that still harbor brGDGTs with similar environmental correlations as soils (Raberg et al., 2022b). Environmentally observed brGDGT patterns could thus be the result of microbial community shifts, the physiological responses of various taxonomic groups, or a combination of both community shifts and physiological responses (De Jonge et al., 2019, 2021; Guo et al., 2022; Raberg et al., 2022a).

As part of ongoing research into the effects of low O_2_ on brGDGT production (e.g., Halamka et al., 2021), we investigated several cultured Acidobacteria that harbor low‐affinity terminal oxidases in their genomes including the SD 3 Acidobacterium *Solibacter usitatus* (Joseph et al., 2003; Ward et al., 2009), hypothesizing that they might be adapted to low O_2_ environments. Here, we report that *S. usitatus* produces the common brGDGTs Ia, IIa, IIIa, Ib, and IIb (Weijers et al., 2007) as well as several other tetraethers including brGTGTs (Glycerol Trialkyl Glycerol Tetraethers) and uncommon isomers of brGDGT IIIa and IIIb. We find that tetraethers comprise a significant fraction of this organism's cellular membrane (24 ± 9% on average across all experimental conditions) and change in relative abundance in response to physiological constraints including temperature, pH, and O_2_, allowing a direct comparison to brGDGT distributions observed in proxy calibration datasets. We demonstrate that the degree of brGDGT methylation in *S. usitatus* in response to temperature variations agrees with empirically developed brGDGT‐based temperature indices. In contrast, we find that the degree of brGDGT cyclization in *S. usitatus* in response to pH does not match environmental trends. Finally, we show that the brGDGT IIIa and IIIb isomers found in *S. usitatus* respond to changes in O_2_.

## 2 MATERIALS AND METHODS

### 2.1 Microbial strains, media, and growth conditions

*Solibacter usitatus* strain Ellin6076 (Joseph et al., 2003, Ward et al., 2009); originally named *Candidatus S. usitatus* but sufficiently described to become a fully established taxon since, (see Oren et al., 2020) was acquired from the German Collection of Microorganisms and Cell Cultures (DSM 22595) and was grown in triplicate in a modified DSMZ 1266 medium at all presented temperature (15, 20, 25, and 30°C), pH (5.0, 5.5, 6.0, 6.5), and oxygen (1%, 5%, 21% O_2_) conditions (see Table S1 for overview). Modified DSMZ 1266 medium consisted of 13.3 mm MES (2‐[*N*‐morpholino] ethanesulfonic acid) buffer, 0.67 g/L yeast extract (YE), 2.5 mm glucose, 0.27 mm MgSO_4_, 0.4 mm CaCl_2_, 0.2 mm KH_2_PO_4_, 0.4 mm NH_4_Cl, 15 nm (3 μg/L) Na_2_SeO_3_, 16 nm (4 μg/L) Na_2_WO_4_, 1.33 ml/L SL10 trace element solution, and 1.33 ml/L HS vitamin solution. SL10 trace element solution contained 1.5 g/L FeCl_2_ × 4 H_2_O, 70 mg/L ZnCl_2_, 100 mg/L MnCl_2_ × 4 H_2_O, 6 mg/L H_3_BO_3_, 190 mg/L CoCl_2_ × 6 H_2_O, 2 mg/L CuCl_2_ × 2 H_2_O, 24 mg/L NiCl_2_ × 6 H_2_O, and 36 mg/L Na_2_MoO_4_ × 2 H_2_O. HS vitamin solution contained 50 mg/L alpha‐lipoic acid (thioctic acid), 50 mg/L biotin (D+), 100 mg/L Ca‐pantothenate (D+), 50 mg/L cyanocobalamin (B12), 50 mg/L folic acid, 100 mg/L nicotinic acid (Niacin), 100 mg/L p/4‐aminobenzoic acid, 100 mg/L pyridoxine hydrochloride, 100 mg/L riboflavin, and 100 mg/L thiamine hydrochloride. Media pH was buffered by MES (pK_a_ 6.15) and adjusted with 5 m NaOH to pH values of 5.0, 5.5, 6.0, and 6.5, depending on the experiment. All pH values were confirmed from media aliquots at the beginning and end of each experiment using a Mettler Toledo InPro4260i pH sensor calibrated with commercial pH 4.0 and 7.0 standards. No significant pH changes were detected from culture growth.

Aerobic culture experiments were conducted in standard yellow‐capped 25 ml culture tubes (18 mm diameter) with 10 ml of media shaken at 250 rotations per minute (rpm) in atmosphere (21% O_2_). Suboxic culture experiments were conducted in 100 ml media bottles with 60 ml of media and gasket‐sealed screw‐cap lids. Suboxic headspace was achieved by continuously flushing the culture headspace through gas‐impermeable 1/8″ PTFE tubing connected to in/out ports with standard ¼‐28 liquid chromatography compression fittings at a rate of 100 ml/min with high purity N_2_ blended with compressed air using digital mass flow controllers (Alicat Scientific, MC‐Series). The gas blend for ~1% O_2_ cultures consisted of 95% N_2_ and 5% air (v/v), and the gas blend for ~5% O_2_ cultures consisted of 75% N_2_ and 25% air (v/v). All suboxic cultures were stirred continuously with a magnetic stir bar at 625 rpm to ensure gas equilibration between headspace and media. The long duration of these growth experiments required that gas was bubbled through sealed sterile water prior to entering the culture vessels to prevent rapid evaporation of culture media. All cultures were incubated in temperature‐controlled forced‐air incubators (capable of heating and cooling) set to 15, 20, 25, and 30°C, depending on the experiment.

To eliminate the risk of contamination by common fast‐growing laboratory contaminants during the long growth experiments (up to 57 days), cultures were never subsampled during growth and growth was monitored exclusively using optical density (OD) measurements through the culture vessels. For aerobic culture tubes, OD measurements were taken manually at 600 nm every 1–4 days using a GENESYS 30 (Thermo Scientific) visible spectrophotometer with adapter for culture tubes. For suboxic culture bottles, OD measurements were recorded automatically every 10 min using a custom 3D‐printed bottle adapter fitted with a narrow beam angle 630 nm LED (Marktech Optoelectronics, MTE7063NK2‐UR) as light source and adjustable gain optical sensor (Texas Instruments, OPT101) as detector controlled by an ARM Cortex M3 powered microcontroller (Particle Industries, PHOTONH). All growth curves are reported in the SI (Figure S2). Growth rates were calculated for all replicate cultures by fitting growth curves to the logistic equation (Table S1, Figure S3). All cultures were inoculated from cells passaged repeatedly in the same medium. Based on optical densities of the inoculum and final optical densities of the batch cultures at harvest, all experiments represent at least 6 generations of cells (Table S1), which means the inoculum contributed maximally 1.6% of the final extracted lipids.

### 2.2 Lipid extraction and analysis

Harvested cells were extracted using the rapid acid hydrolysis‐methanolysis protocol described in Halamka et al. (2021). Briefly, cells from liquid culture were harvested in stationary phase by centrifugation (5000 rpm for 3 min). Harvested cells were lyophilized overnight and then physically disrupted in 2 ml microcentrifuge tubes by vortexing with 250 μl methanol (MeOH) and 250 μl volume equivalent of 100 μm muffled glass beads for 10 min at 3000 rpm using a Disruptor Genie (Scientific Industries, SI‐DD38). Excess MeOH was evaporated and 25 μg of 23:0 PC (1,2‐ditricosanoyl‐sn‐glycero‐3‐phosphocholine), 25 μg of 24:0 fatty acid (tetracosanoic acid), and 25 ng of C46 GTGT (glycerol trialkyl glycerol tetraether; Huguet et al., 2006) were added to all samples as internal quantification standards. Lipids were extracted for 90 min at 65°C with 500 μl 3 N hydrochloric acid (HCl) in MeOH (33% final water content) to cleave headgroups and transesterify fatty acid esters to fatty acid methyl esters (FAMEs). Samples were cooled for 10‐min before the addition of 500 μl methyl tert butyl ether (MTBE). The upper organic phase was extracted 3 times with 500 μl *n*‐hexane, and total lipid extracts (TLEs) were evaporated under N_2_.

Monoacyl glycerol ethers (MAGEs, or monoethers) and diacyl Glycerol Ethers (DAGEs, or diethers) were acetylated for gas chromatography (GC) analysis by suspending in 100 μl of dichloromethane (DCM) with the addition of 20 μl of anhydrous pyridine and 20 μl of acetic anhydride. Samples were then incubated at 70°C for 20 min before evaporation and resuspension in *n*‐hexane for analysis. FAMEs, MAGEs, and DAGEs were analyzed in the CU Boulder Earth Systems Stable Isotope Lab on a Thermo Trace 1310 GC using a SSL injector and a 30 m DB‐5 HT capillary column (Agilent Technologies, 0.25 mm I.D., 0.25 μm film thickness; 2 min at 40°C, ramped to 295°C at 15°C/min, ramped to 315°C at 5°C/min, and ramped to 375°C at 15°C/min then held for 5 min at 375°C). Compounds were identified based on retention times compared with a bacterial acid methyl ester (BAME) standard (Millipore Sigma) and a 37 FAME standard (Supelco) and by their characteristic fragmentation patterns using a Thermo Scientific ISQ Single Quadrupole Mass Spectrometer on full scan mode and quantified by flame ionization detector (FID) in comparison with the 24:0 fatty acid extraction standard.

Tetraethers were analyzed in the Organic Geochemistry Laboratory at the University of Colorado Boulder on a Thermo Scientific Ultimate 3000 high‐performance liquid chromatograph (HPLC) coupled to a Q Exactive Focus Orbitrap‐Quadrupole MS with an atmospheric pressure chemical ionization (APCI) source using a previously published normal phase (NP) method (Hopmans et al., 2016) with the following modifications to slightly lengthen the method for isomer separation (Raberg et al., 2021): the initial isocratic elution used 14% instead of 18% eluent B (9:1 hexane: isopropanol) and correspondingly 86% instead of 82% eluent A (hexane). This 25‐min isocratic hold was followed by a linear gradient to 35% B over 35 min instead of over 25 min (Hopmans et al., 2016); then, a linear gradient to 100% B over 30 min (same as Hopmans et al., 2016), re‐equilibration to 14% B over 1 minute, and ending on a 19‐min isocratic hold at 14% B for a total of 110 min of run time with a flow rate of 0.2 ml/min. BrGDGTs were identified using their corresponding retention times in an in‐house environmental reference sample (0–1 cm surface sediment from lake 3LN, northern Quebec, Raberg et al., 2021), their molecular masses, and the MS/MS spectra generated by data‐dependent acquisition mode (ddMS/MS). A subset of TLE samples was also analyzed by reverse phase (RP) LC (Connock et al., 2022) to confirm relative elution order of brGDGT isomers and further constrain their identity (data not shown). Cellular tetraether abundances were calculated relative to fatty acids (FA) and mono/diethers using the C24 and C46 internal standards. Tetraether abundances relative to the standard brGDGTs are reported as %br:

(1) $$\%\mathrm{br}=\left[\left(\text{brGDGT}\;x\right)/\left(\mathrm{Ia}+\mathrm{Ib}+\mathrm{Ic}+\mathrm{IIa}+\mathrm{IIb}+\mathrm{IIc}+\text{IIIa}+\text{IIIb}+\text{IIIc}\right)\right]\times 100$$

### 2.3 Environmental samples

To test culture production of brGDGTs against brGDGT‐based proxy calibration datasets, we compare brGDGTs produced by *S. usitatus* against a comprehensive compilation of brGDGT observations in environmental samples (Raberg et al., 2022b). The compiled dataset includes global observations from the six sample types most often reported on in brGDGT literature: soil, peat, lacustrine sediment, lacustrine settling/suspended particulate matter (SPM), marine sediment, and bone. Reported temperatures for environmental samples were standardized where possible by Raberg et al. (2022a, 2022b) but are necessarily++ different for some sample types (e.g., water temperature for lacustrine SPM versus air temperature for bone). To further supplement the dataset presented by Raberg et al. (2022b), we compiled soils published with in situ soil temperature data (recorded using temperature loggers at 2–10 cm depth) from Wang et al. (2020), Pérez‐Angel et al. (2020), De Jonge et al., 2019, Sigurdsson et al. (2016), Wang and Liu (2021), and Halffman et al. (2022). We used mean monthly temperatures, either calculated from hourly measurements (De Jonge et al. 2019; Sigurdsson et al. 2016; May 8, 2013, to May 7, 2015), provided through personal communication (H. Wang and W. Liu, September 3, 2020; Wang et al. 2020), or reported (Pérez‐Angel et al., 2020), to calculate in situ mean annual temperature (MAT), mean temperature of months above freezing (MAF), mean summer (June, July, and August) temperature (JJA), and temperature of the warmest month (WMT). For all other sites, we used reported in situ temperature parameters as available. One soil (site 5F of De Jonge et al. 2019) was removed as an outlier (residual >3* RMSE of linear regressions between MBT′_5Me_ and temperature for all in situ temperature parameters).

### 2.4 Branched GDGT Indices and Statistics

The brGDGT indices MBT′_5Me_ (De Jonge, Hopmans, et al., 2014), CBT_5Me_ (De Jonge, Hopmans, et al., 2014), and degree of cyclization (DC; Baxter et al., 2019) were calculated as follows:

(2) $$\mathrm{MB}T_{5\mathrm{Me}}^{\prime }=\left(\mathrm{Ia}+\mathrm{Ib}+\mathrm{Ic}\right)/\left(\mathrm{Ia}+\mathrm{Ib}+\mathrm{Ic}+\mathrm{IIa}+\mathrm{IIb}+\mathrm{IIc}+\text{IIIa}\right)$$

(3) $${\mathrm{CBT}}_{5\mathrm{Me}}=-\log\left(\left(\mathrm{Ib}+\mathrm{IIb}\right)/\left(\mathrm{Ia}+\mathrm{IIa}\right)\right)$$

(4) $$\mathrm{DC}=\left(\mathrm{Ib}+2^{*}\;\mathrm{Ic}+\mathrm{IIb}+\mathrm{II}b^{\prime }\right)/\left(\mathrm{Ia}+\mathrm{Ib}+\mathrm{Ic}+\mathrm{IIa}+\mathrm{II}a^{\prime }+\mathrm{IIb}+\mathrm{II}b^{\prime }\right)$$

BrGDGT fractional abundances in the methylation and cyclization sets were calculated according to Raberg et al. (2021). Broadly, for brGDGT with roman numeral *x* (I, II, or III) and letter *y* (a, b, or c), the fractional abundance *f* in a given structural set *S* is calculated as follows:

(5) $${\mathrm{fxy}}_{S}=\mathrm{xy}/\sum \left(\text{brGDGTs in}\;S\right).$$

Correlations between the calculated MBT′_5Me_ and CBT_Me_ indices and the culture and environmental data were evaluated using Pearson correlation coefficients (*r*) and their *p*‐values. For culture data from this study, the growth parameters temperature, growth rate, pH, and % O_2_ were evaluated. For environmental data, the environmental parameters temperature and pH were evaluated, as well as the in situ temperatures discussed above. For statistically significant correlations (*p* < .05), the regression coefficients (slope and intercept) and coefficient of determination (*R* ^2^) were calculated using linear least squares fits of the index versus parameter. Coincidence between linear regressions for the different environmental datasets and the culture data was evaluated using the following dummy variable regression model (Clogg et al., 1995):

(6) $$\begin{array}{c} \text{index}=\text{intercept}+\Delta \text{intercept}\cdot \left[\text{culture}\right]+\text{slope}\cdot \text{parameter}\\ \;+\Delta \text{slope}\cdot \left[\text{culture}\right]\cdot \text{parameter} \end{array}$$

with [culture] representing the dummy variable (0 for environmental data, 1 for culture data), and Δintercept and Δslope representing the intercept and slope differences between the culture and environmental data sets. Significance of the regression fit differences was evaluated using the p‐values of the Δintercept and Δslope coefficients. Δintercept values that are not statistically different from 0 (Δintercept *p* > .05) indicate concurrent culture and environmental data (i.e., they have the same intercept); Δslope values that are not statistically different from 0 (Δslope *p* > .05) indicate parallel culture and environmental data (i.e., they have the same slope); comparisons where both is the case indicate coincident culture and environmental data (i.e., they have the same intercept and slope and are therefore statistically indistinguishable regression fits).

## 3 RESULTS

*Solibacter usitatus* grew successfully under all tested conditions (Figure S1) except for pH 6.5 at 15°C. The specific growth rate ranged from 0.23 day^−1^ (doubling time: 3.0 days) at pH 6.0, 15°C and 21% O_2_ to 1.45 day^−1^ at pH 5.5, 30°C and 21% O_2_ (doubling time: 11.5 h). Growth rates increased systematically from low‐to‐high growth temperatures and decreased systematically from high‐to‐low O_2_ (Table S1, Figure S2). *Solibacter usitatus* produced a range of saturated, monounsaturated, and terminally methyl‐branched FA, as well as several mono‐ (MAGE) and diether (DAGE) glycerols with iso‐C15:0 FA, iso‐C17:1 FA, iso‐C15:0 DAGE, and iso‐C15:0 MAGE the most abundant components (data in Tables S2 and S3; structures in Table S7). However, *S. usitatus* produced no iso‐diabolic acid (13,16‐dimethyl octacosanedioic acid) or its mono‐glycerol‐bound equivalents in agreement with the results of previous culture work on this organism (Sinninghe Damsté et al., 2018). In addition, we detected a wide range of tetraethers under all growth conditions including brGTGTs and brGDGTs comprising an estimated 10%–47% of the cellular membrane (mean = 24.0%, 1σ = 9%, Tables S2 and S4). Whereas recent work on another Acidobacterium in culture, *Edaphobacter aggregans*, demonstrated brGDGT production, was tied to low oxygen growth conditions (Halamka et al., 2021), brGDGTs were abundant in *S. usitatus* at both low and high oxygen concentrations and were most abundant overall at lower pH, lower temperature, and lower O_2_ (Figure S3).

**FIGURE 1:**
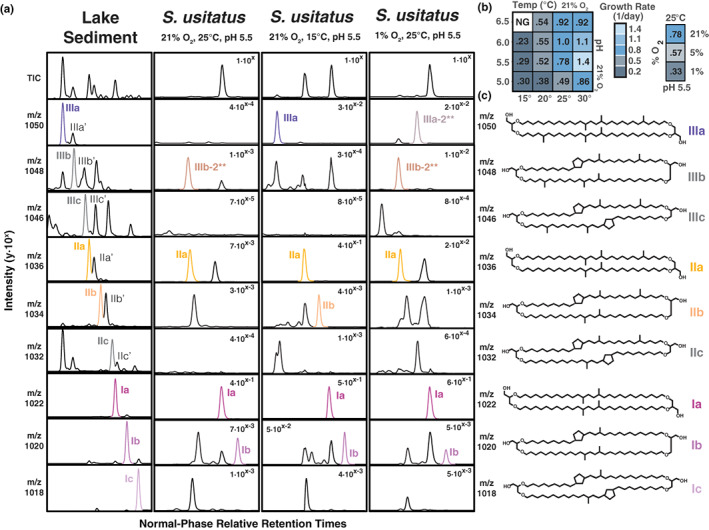
Mass channel extracted chromatographs, culturing growth rates, and brGDGT structures of *Solibacter usitatus* and an environmental reference sample. (a) Normal phase total ion chromatogram (TIC) and selected mass channels showing data from the environmental sample (0–1 cm surface sediment from lake 3LN, northern Quebec) and three culturing conditions of *S. usitatus*. Chromatographic window spans from brGDGT IIIa to brGDGT Ic and retention times are relative to brGDGT Ia within each sample. Peak intensities are normalized to TIC intensity within each sample and shown as y⋅10× in each panel. BrGDGT peaks are color‐coded with their corresponding structure labels in (c). (b) Overview of all culturing conditions analyzed for this study with averaged growth rates (1 per day) of biological triplicates. (c) Structures of the most common brGDGTs with additional methylations at C5. Structures for common isomers not observed in *S. usitatus* culture (e.g., IIIa/b/c′, IIa/b/c′) are not shown in this figure (see Table S7 instead). Structures for the uncommon isomers IIIa‐2 and IIIb‐2 (*) are shown in Figure 3

**FIGURE 2:**
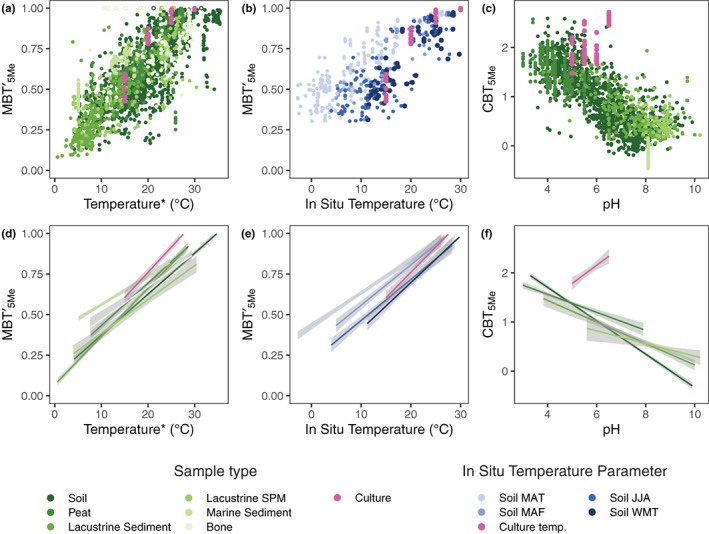
Relationships between primary brGDGTs and temperature and pH for *Solibacter usitatus* cultures and environmental samples. (a) Relationship between the MBT′_5Me_ index and temperature for cultures (pink) and environmental samples (green; SPM = suspended/settling particulate matter). *Temperatures were associated with environmental samples following Raberg et al. (2022a); see Dataset S2 for details. Sample type‐specific linear slopes are provided in (d) with 95% confidence intervals in gray. Samples with MBT′_5Me_ = 1 were considered outliers and are plotted as open circles in (a) and removed from linear fits in d). (b) Relationship between the MBT′_5Me_ index and in situ temperatures for soils (blue) and cultures (pink). Shades of blue represent in situ soil temperatures averaged over different portions of the year, with abbreviations as follows: mean annual temperature (MAT), mean temperature of months above freezing (MAF), mean summer (June, July, and August) temperature (JJA), and warmest month temperature (WMT). Sample type/temperature parameter‐specific linear regressions are plotted in (e). (c) Relationship between CBT_5Me_ and pH for cultures and environmental samples, with sample type‐specific slopes plotted in (f). Regression coefficients and statistics for d–f are provided in Table 1

Five of the 15 commonly studied brGDGTs—Ia, IIa, IIIa, Ib, and IIb—were abundant in *S. usitatus* cultures (Figure 1, Tables S4 and S5). Mass traces of brGDGTs IIIb, Ic, IIc, and IIIc were also identified in some cultures, but at exceedingly low abundances close to the detection limit and thus not quantified further. Although 6‐methyl isomers of penta‐methylated and hexa‐methylated brGDGTs (IIa′, IIb′, IIc′, IIIa′, IIIb′, and IIIc′) are common in environmental samples (De Jonge et al., 2013; De Jonge, Hopmans, et al., 2014), the isomers detected in *S. usitatus* (IIa, IIb, and IIIa) were all methylated at the C5 position. The resulting branched brGDGT indices MBT′_5Me_ and CBT_5Me_ were calculated for all culture conditions (Table S5). MBT′_5Me_ showed statistically significant positive correlations (*p* < 0.05) with culture temperature and growth rate across all data, as well as negative correlations with pH for cultures grown at 15 and 20°C (Table 1, Figure S4). CBT_5Me_ was significantly correlated only with culture pH (Table 1). The brGDGT responses to growth rate, temperature, pH, and O_2_ in culture, as well as comparisons with environmental data (Figure 2), are detailed in the sections below.

**TABLE 1:** Pearson correlation coefficients (a), linear regression fits (b), and statistical comparisons between culture and environmental data (c) for MBT′_5Me_ and CBT_5Me_ indices calculated from *Solibacter usitatus* culture data and environmental samples.

Dataset		Parameter	Pearson corr. (A)		Linear regression (B)			Difference to culture data (C)			
			*r*	Signif	Slope	Intercept	*R* ^2^	Δslope	Signif	Δintercept	Signif
*Index: MBT′*_*5Me*_											
*S. usitatus* Culture Data	All data	Temperature	**+0.91**	<0.001 (***)	0.031 ± 0.002	0.14 ± 0.05	0.82				
	All data	Growth rate (1 per day)	**+0.68**	<0.001 (***)	0.347 ± 0.054	0.63 ± 0.04	0.45				
	30°C subset		**+0.72**	<0.01 (**)	0.007 ± 0.002	0.99 ± 0.00	0.48				
	25°C subset		+0.43	0.08 (—)							
	20°C subset		**−0.78**	<0.01 (**)	−0.289 ± 0.073	0.95 ± 0.04	0.57				
	15°C subset		+0.47	0.20 (—)							
	All data	pH	+0.06	0.69 (—)							
	30°C subset		+0.48	0.11 (—)							
	25°C subset		+0.31	0.22 (—)							
	20°C subset		**−0.88**	<0.001 (***)	−0.046 ± 0.008	1.08 ± 0.05	0.75				
	15°C subset		**−0.93**	<0.001 (***)	−0.139 ± 0.021	1.29 ± 0.11	0.85				
	O_2_ subset	% O_2_	+0.53	0.14 (—)							
Environmental Data	Bone	WMT (air)	**+0.74**	<0.001 (***)	0.020 ± 0.002	0.27 ± 0.05	0.54	0.011 ± 0.003	<0.01 (**)	−0.13 ± 0.08	0.11 (—)

Lacustrine Sed.	MAF (air)	**+0.89**	<0.001 (***)	0.030 ± 0.001	0.07 ± 0.01	0.79	0.001 ± 0.003	**0.61 (—)**	0.07 ± 0.06	**0.27 (—)**

Lacustrine SPM	Water temp.	**+0.76**	<0.001 (***)	0.021 ± 0.002	0.18 ± 0.03	0.57	0.010 ± 0.004	<0.01 (**)	−0.04 ± 0.09	0.68 (—)

Marine sediment	SST	**+0.89**	<0.001 (***)	0.017 ± 0.001	0.39 ± 0.01	0.78	0.014 ± 0.002	<0.001 (***)	−0.25 ± 0.04	<0.001 (***)

Peat	WMT (air)	**+0.78**	<0.001 (***)	0.026 ± 0.001	0.17 ± 0.02	0.61	0.005 ± 0.003	**0.14 (—)**	−0.03 ± 0.08	**0.68 (—)**

Soil	WMT (air)	**+0.77**	<0.001 (***)	0.025 ± 0.001	0.12 ± 0.01	0.59	0.006 ± 0.004	**0.11 (—)**	0.02 ± 0.09	**0.84 (—)**

	In situ WMT	**+0.87**	<0.001 (***)	0.029 ± 0.002	0.12 ± 0.04	0.75	0.002 ± 0.003	**0.45 (—)**	0.02 ± 0.07	**0.80 (—)**

	In situ JJA	**+0.82**	<0.001 (***)	0.025 ± 0.002	0.21 ± 0.03	0.67	0.006 ± 0.003	**0.07 (—)**	−0.07 ± 0.07	**0.31 (—)**

In situ MAF	**+0.88**	<0.001 (***)	0.025 ± 0.002	0.31 ± 0.03	0.78	0.006 ± 0.003	<0.05 (*)	−0.17 ± 0.06	<0.01 (**)

In situ MAT	**+0.85**	<0.001 (***)	0.021 ± 0.001	0.43 ± 0.01	0.71	0.011 ± 0.003	<0.001 (***)	−0.29 ± 0.06	<0.001 (***)
*Index: CBT*_*5Me*_											
Culture	All data	Temperature	−0.10	0.50 (—)							
	All data	Growth rate	−0.21	0.14 (—)							
	All data	pH	**+0.56**	<0.001 (***)	0.37 ± 0.08	−0.1 ± 0.4	0.30				
	O_2_ subset	% O_2_	−0.64	0.06 (—)							
Env.	Lacustrine Sed.	pH	**−0.59**	<0.001 (***)	−0.22 ± 0.02	2.3 ± 0.2	0.34	0.59 ± 0.09	<0.001 (***)	−2.4 ± 0.5	<0.001 (***)
	Lacustrine SPM	pH	**−0.32**	<0.01 (**)	−0.13 ± 0.04	1.6 ± 0.4	0.09	0.50 ± 0.08	<0.001 (***)	−1.6 ± 0.6	<0.01 (**)
	Peat	pH	**−0.46**	<0.001 (***)	−0.18 ± 0.02	2.3 ± 0.1	0.21	0.55 ± 0.09	<0.001 (***)	−2.3 ± 0.5	<0.001 (***)
	Soil	pH	**−0.77**	<0.001 (***)	−0.34 ± 0.01	3.1 ± 0.1	0.59	0.71 ± 0.10	<0.001 (***)	−3.1 ± 0.6	<0.001 (***)

*Note*: Environmental datasets and parameters correspond to those shown in Figure 2. (A) Statistically significant correlation coefficients (*r*) are highlighted in bold (*p* < .05). (B) All regression coefficients are shown including ±1 standard error. All temperatures are in °C with slopes versus temperature in index/°C. (C) Statistical comparison between the *Solibacter usitatus* culture data and different environmental data sets was conducted as outlined in Section 2. Differences in slope (Δslope) and intercept (Δintercept) that are not statistically significant (coefficient *p* > .05) are highlighted in bold and indicate that culture and environmental data is concurrent (Δintercept ≈0) and/or parallel (Δslope ≈0). Comparisons where both is the case (concurrent and parallel) indicate that the compared culture and environmental regressions are statistically indistinguishable. See Dataset S2 for all data in spreadsheet format

O_2_ subset includes culture data at 1%, 5%, and 21% O_2_ all at 25°C, pH 5.5.

*p*‐value significance levels (***<.001; **<.01; *<.05; —, not significant).

In addition to the above brGDGTs, *S. usitatus* produced brGTGT equivalents of brGDGT Ia and IIa (Figure S5) with brGTGT Ia making up a significant portion of the detected tetraethers across all culture conditions (between 1.3% and 9.9%, Table S4). *Solibacter usitatus* also produced variable quantities (0.11%–3.8% of detected tetraethers) of a brGTGT with the same molecular mass as brGTGT IIIa but a combination of C30, C15, and C17 alkyl chains instead of C31, C15, and C16 expected in symmetric brGTGT IIIa (Figure S5). We propose that the unusual C17 alkyl chain of this brGTGT (hereafter brGTGT IIIa‐2) could be overly branched with a methyl group at C9 in addition to the common brGDGT methylations at C5 and C13 (see structure in Figure S5). Alternatively, the C17 alkyl chain could be elongated (e.g., iso‐C17:0 or straight C17:0) instead given the production of the corresponding FAs by *S. usitatus* (Table S3).

**FIGURE 3:**
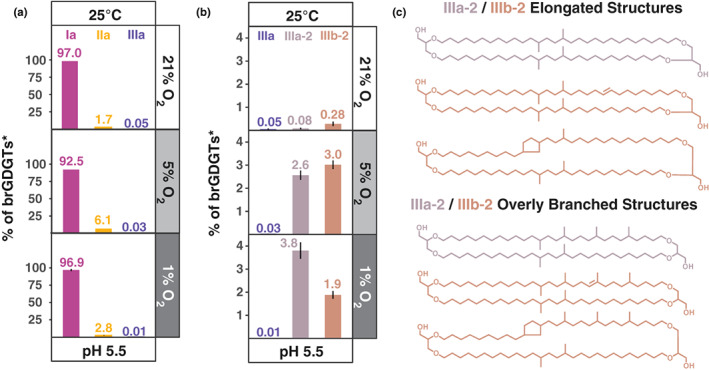
Influence of oxygen concentration on brGDGT production in *Solibacter usitatus*. (a) Relative abundance (%br) of brGDGT Ia (magenta), brGDGT IIa (orange), and brGDGT IIIa (purple) at all tested oxygen concentrations. (b) Relative abundance (%br) of brGDGT IIIa (purple), brGDGTIIIa‐2 (light purple), and brGDGT IIIb‐2 (brick) at all tested oxygen concentrations. (c) Proposed potential structures for brGDGT IIIa‐2 and brGDGT IIIb‐2. *See Equation 1 for %br calculation: brGDGT IIIa‐2 and brGDGT IIIb‐2 are not included in the denominator

Lastly, *S. usitatus* produced two uncommon brGDGT isomers in significant quantities up to 3.5%/2.7% of detected tetraethers (Table S4) or 3.8%br/3.0%br relative to the common brGDGTs (Figure 3, Table S5) that showed a strong response to oxygen limitation. These isomers have identical molecular masses to the hexamethyl brGDGTs IIIa and IIIb, respectively (Figure S5), and are referred to as brGDGT IIIa‐2 and brGDGT IIIb‐2 accordingly (Figure 1). Both IIIa‐2 and IIIb‐2 are asymmetrical tetraethers composed of a C30 alkyl chain and a C32 alkyl chain that has one unsaturation in the case of IIIb‐2. Both were chromatographically resolved from brGDGT IIIa/IIIb (Figure S6) suggesting that they cannot be methylated at the C5 position like IIIa/IIIb since asymmetric brGDGTs are not readily distinguishable by liquid chromatography from their symmetric counterparts (De Jonge et al., 2013; Weber et al., 2018).

With the normal phase HPLC method used in this study, brGDGT IIIa‐2 eluted 3.55 ± 0.10 min later than IIIa with chromatographic resolution of 7.5 ± 0.2 (Hopmans et al., 2016; Snyder et al., 1997). The compound further eluted an estimated 2.7 min later than IIIa’/IIIa_6_ (methylated at C6), and an estimated 1.7 min later than the IIIa_7_ isomer (methylated at C7, Ding et al., 2016). IIIa′/IIIa_6_ and IIIa_7_ were not present in the culture samples, and their retention times were compared from environmental samples analyzed with the same method (see chromatograms in Figure S6). These observations suggest that brGDGT IIIa‐2 could have an unusual methylation pattern that leads to NP elution significantly later than the IIIa/IIIa_5_, IIIa′/IIIa_6,_ and IIIa_7_ isomers. Based on the existence of the above described brGTGT IIIa‐2 with its unusual C17 alkyl chain (Figure S5), it is conceivable that brGDGT IIIa‐2 has an equivalent structure with a C32 alkyl‐chain either overly branched or asymmetrically elongated. We thus propose two potential structures for brGDGT IIIa‐2 (Figure 3) that fit our data, one a hexamethyl brGDGT that is asymmetrically methylated at C5 and C9, and the other an elongated tetramethyl brGDGT with iso‐C17:0 as a precursor.

BrGDGT IIIb‐2 eluted 2.19 ± 0.03 min later than IIIa with the NP‐HPLC method (chromatographic resolution 4.6 ± 0.1), 1.3 min later than the expected elution time of IIIb and between IIIb′/IIIb_6_ and IIIb_7_, but 1.4 min earlier than brGDGT IIIa‐2 (Figure S6). We hypothesize that brGDGT IIIb‐2 is either the monocyclic or the monounsaturated equivalent of brGDGT IIIa‐2, with the elution time and abundance of iso‐C17:1 in *S. usitatus* (Table S3) pointing to the asymmetrically elongated structure with a double bond the most likely. Future analyses of purified concentrated fractions of brGTGT IIIa‐2, brGDGT IIIa‐2, and brGDGT IIIb‐2 using GC–MS after ether cleavage or NMR will help determine the exact structure of these isomers. Lastly, several other uncharacterized ether lipids were detected in *S. usitatus* by NP and/or RP HPLC‐MS but in minor quantities. Their structural characterization was beyond the scope of this paper and will be the subject of future work.

### 3.1 BrGDGT response to growth rate

The brGDGT distribution of *S. usitatus* changed with growth rate (Figure S4) with the MBT′_5Me_ index positively correlated across all culture data (*r* = .68, *p* < .001, Table 1). However, growth rate was also strongly correlated with growth temperature (*r* = .79, *p* < .001). In a multivariable regression of MBT′_5Me_ vs temperature and growth rate, growth rate was not a significant predictor (*p* > .2) with temperature alone explaining 82% of the variance in the data. Furthermore, the MBT′_5Me_ index within temperature subsets showed inconsistent correlations with growth temperature ranging from positively correlated at 30°C (*r* = .72, *p* <.01) to the opposite anticorrelation at 20°C (*r* = −.78, *p* < .01, Table 1, Figure S4). This suggests that growth rate is not the primary mechanistic driver of brGDGT distributions in *S. usitatus*, although rate‐controlled experiments in continuous culture at fixed temperatures will be required to fully deconvolute its effects in this organism.

### 3.2 BrGDGT response to temperature

Growth temperature was the most impactful of the tested growth parameters. A significant increase in methylation number, as captured by both the MBT′_5Me_ index (*r* = .91, *p* < .001, Table 1a; Figure 2a,b,d,e; Figure S4) and the Meth Set fractional abundances (Equation 5; Figures S7 and S8), was observed at colder temperatures. The increases in methylation number occurred in parallel in acyclic and monocyclic brGDGTs (Figure S8); fIa_Meth_ and fIb_Meth_ had a one‐to‐one correlation (slope = 1.00 ± 0.03) with *R* ^2^ = 0.94 (*p* < .001) across all culturing conditions. MBT′_5Me_ and meth set temperature relationships in *S. usitatus* were overall in good agreement with relationships observed in the wide range of environmental sample types included in the compiled environmental dataset, including soils, peats, lacustrine sediments and SPM, marine sediments, and bone (Figure 2a,d, Figures S8 and S9). Of these sample types, regressions of the MBT′_5Me_ index vs. temperature for the *S. usitatus* culture data were indistinguishable from environmental data sets for lacustrine sediments (vs MAF air), peats (vs WMT air), and soils (vs WMT air) with slope and intercept differences (Δ slope, Δ intercept) statistically indistinguishable from 0 (*p* > .1, Table 1c). For in situ soil data, the culture data were in strongest agreement with MBT′_5Me_ regression vs WMT (warmest month temperature) with slope/intercept differences of 0.002 ± 0.003°C^−1^ ≈ 0 (*p*‐value >0.4)/0.02 ± 0.07 ≈ 0 (*p* > .8) followed by regression versus JJA (summer months temperature) with slope/intercept differences of 0.006 ± 0.003°C^−1^ (*p*‐value >0.07)/−0.07 ± 0.07 ≈ 0 (*p* > .3). The inclusion of in situ temperatures from colder months led to statistically significant differences from the culture data, with regression vs. MAF (months above freezing temperature) producing moderately significant slope/intercept differences (Δ slope = 0.006 ± 0.003°C^−1^, Δ intercept = −0.17 ± 0.06, *p* <.05), and regression vs MAT producing highly significant differences (Δ slope = 0.011 ± 0.003°C^−1^, Δintercept = −0.29 ± 0.06, *p* < .001) from the culture data (Table 1c).

Although the methylation of brGDGTs in *S. usitatus* was most affected by temperature, pH was also observed to have a significant impact but only at lower temperatures, not across the entire data set (Figure S4). Specifically, MBT′_5Me_ values were significantly anticorrelated with pH for *S. usitatus* growth at 20 and 15°C, with correlation coefficients of −0.88 and −0.93 (*p* < .001, Table 1a), respectively, reflecting a higher degree of brGDGT methylation with higher pH at the same temperature.

### 3.3 BrGDGT Response to pH

In addition to its effects on brGDGT methylation at lower temperature, pH affected the cyclization of brGDGTs in *S. usitatus*. pH was the only growth parameter that showed a significant correlation with the CBT_5Me_ index (*r* = .56, *p* < .001, Table 1a). However, the observed effect on CBT_5Me_ was nearly orthogonal to the strong anticorrelation typically observed in environmental samples (Figure 2c,f, Table 1a). Examination of the Cyclization Set fractional abundances revealed that this increase in CBT_5Me_ with pH was driven by decreasing relative abundances of cyclized compounds (Ib and IIb; Figure S10). However, we note that these fractional abundance decreases were slight (magnitude of linear slopes <0.7%/pH unit; Figure S11) and that *S. usitatus* cultures generally plotted within the scatter of environmental samples (Figure S10). Due to its logarithmic formulation, CBT_5Me_ is highly sensitive when the degree of brGDGT cyclization is small, as was the case for *S. usitatus* cultures (Figure S12). Therefore, the departure in the CBT_5Me_ index may overemphasize minor changes across a limited gradient that would be less meaningful if tested across a broader range of pH. We were unsuccessful in growing *S. usitatus* outside of the 5.0–6.5 pH range to further test this hypothesis.

### 3.4 BrGDGT response to oxygen limitation

Due to experimental limitations, the brGDGT response in *S. usitatus* was only tested at three levels of O_2_ in the headspace (21%, 5%, and 1% O_2_) at one pH and temperature (pH 5.5 and 25°C). The results showed no systematic correlations of the MBT’_5Me_ and CBT_5Me_ indices with O_2_ (Table 1a), but brGDGT abundances at 5% and 1% O_2_ still showed several differences in brGDGT methylation compared with the fully oxygenated (21% O_2_) condition (Figure 3a). Specifically, an increase in brGDGT IIa was observed at 5% O_2_ (6.1 ± 0.2%br) relative to atmospheric O_2_ (1.68 ± 0.02%br) coupled to a decrease in brGDGT Ia (92.5 ± 0.1%br at 5% O_2_ compared with 97.0 ± 0.1%br at 21% O_2_). At 1% O_2_, brGDGT Ia was identical within error to its relative abundance at 21% O_2_ (96.9 ± 0.3%br). Despite the similar dominance of brGDGT Ia at 1% O_2_ and 21% O_2_, an increase in the proportion of brGDGT IIa to 2.8 ± 0.3%br (vs. 1.68 ± 0.02%br at 21% O_2_) and a decrease in the percentage of brGDGT IIIa to 0.01% (vs. 0.05 ± 0.03%br at 21% O_2_) were observed. These differences led to small changes in the resulting MBT’_5Me_ index from 0.98 at 21% O_2_ to 0.94 and 0.97 at 5 and 1% O_2_, respectively (Table S5, Figure S4).

However, the uncommon brGDGTs IIIa‐2 and IIIb‐2 (Figure 3b, Figure S5) were strongly anticorrelated with %O_2_ (*r* = −.99 and −0.93; *p* < .001). BrGDGT IIIa‐2 increased from near‐zero abundance (0.08 ± 0.04%br) at 21% O_2_ to 2.6 ± 0.2%br at 5% O_2_ and 3.8 ± 0.4%br at 1% O_2_ while brGDGT IIIb‐2 increased from near‐zero abundance (0.3 ± 0.1%br) at 21% O_2_ to 3.0 ± 0.2%br at 5% O_2_ and 1.9 ± 0.2%br at 1% O_2_ (Figure 3b).

## 4 DISCUSSION

### 4.1 Environmental relevance

Uncultured strains of *S. usitatus* and other presently uncultured Acidobacteria with a high degree of genetic similarity to *S. usitatus* are abundant in Antarctic and Arctic soils (Mannisto et al., 2007; Pearce et al., 2012). This suggests that the *S. usitatus* strain (Ellin6076) studied here is a relevant model organism for at least one group of potential brGDGT producers, although a single cultured strain from a single species is certainly unlikely to be representative of all environmentally relevant brGDGT producers. *Solibacter usitatus* also provides an interesting case study for understanding the purpose of brGDGT production in cellular membranes. The unique properties and size of the genome of *S. usitatus* provide insights into the functional modalities of this brGDGT‐producing species in the environment (Challacombe et al., 2011; Ward et al., 2009). *Solibacter usitatus* has a 9.9 Mb genome, approximately 2–5 times as large as other sequenced Acidobacteria genomes, and the most Sigma E homologs identified in any sequenced bacterium (Challacombe et al., 2011). Sigma E regulons in bacteria have been attributed to cellular stress responses such as nutrient limitation, oxidative stress, heat shock, and cellular envelope stress in addition to activating outer membrane synthesis and assembly (Challacombe et al., 2011; Kenyon et al., 2005; Raivio & Silhavy, 2001; Rhodius et al., 2005). These genomic properties agree with the general consensus that many SD 1 and 3 Acidobacteria are robust oligotrophs that may have selective advantages in times of stress (Eichorst et al., 2018). The physiological response of brGDGT methylation number to temperature in *S. usitatus* provides insights into the competitive advantage that brGDGTs may provide to oligotrophic bacteria.

### 4.2 Implications for brGDGT‐based paleoenvironmental proxies

The data from *S. usitatus* show that relationships between brGDGTs and temperature observed widely in the environment can be reproduced by a single bacterial species in culture (Figure 2a,d). Although it is important to use caution when extrapolating results from *S. usitatus* to the global environment, several implications for brGDGTs as a paleotemperature proxy are noteworthy.

First, the membrane restructuring exhibited by *S. usitatus* in response to temperature change supports the hypothesis that methylation number plays an important role in membrane homeoviscosity, as suggested by early analogies to other lipid classes (Weijers et al., 2007) and recent molecular dynamics simulations (Naafs et al., 2021). Second, the co‐occurrence of all major brGDGT methylation numbers in *S. usitatus* provides support for the hypothesis that physiological adaptations of a limited group of brGDGT producers could be responsible for the distribution of the major methylated varieties of brGDGTs in the environment as opposed to resulting solely from microbial community shifts.

Alterations to methylation number in *S. usitatus* occurred in acyclic and monocyclic compounds in tandem (*R* ^2^ = 0.94; Figure S8), further suggesting that either the enzyme responsible for C5 methylation indiscriminately methylates acyclic and monocyclic brGDGTs alike and/or that brGDGT cyclases function independently of existing C5 methylations, as has been suggested from observations in environmental samples (Raberg et al., 2021, 2022a). The fact that these temperature‐driven variations in brGDGT distributions are mirrored in a wide array of environmental samples may suggest that the physiological basis for trends observed in *S. usitatus* is widespread in nature, lending confidence to the application of brGDGT‐based paleotemperature proxies and encouraging their further development. For environmental soils in particular, in situ temperatures from the warmest months of the year produced the closest agreement with trends observed in culture (Table 1, Figure 2b,e). We also observed that the growth rate of *S. usitatus* was temperature‐dependent, with a roughly fourfold increase in growth rate when temperature was raised from 15 to 30°C (Figure 1b, Figure S2). Growth rate was similarly observed to be temperature‐dependent in lacustrine microcosm incubations (Martínez‐Sosa et al., 2020). Taken together, these observations suggest that the observed warm‐season bias in empirical calibrations (e.g., Dearing Crampton‐Flood et al., 2020) may originate from seasonal differences in bacterial growth rates with increased production of brGDGTs in warm summer months, a result that may help to guide future proxy calibration approaches.

At the same time, it is important to note the need for cold‐adapted culture isolates and isolates from other environments that produce brGDGTs. The strain of *S. usitatus* studied here (Ellin6076) was isolated from soil in a temperate climate (Victoria, Australia; Joseph et al., 2003). It does not grow reliably at temperatures below 15°C, neutral or alkaline pH, and does not represent freshwater or marine environments. This necessarily precludes studies with this particular strain from capturing the full range of environments where brGDGTs have been observed, particularly cold environments like the Arctic and Antarctic where brGDGTs are frequently employed in paleoenvironmental reconstructions. Additionally, *S. usitatus* was not observed to produce the 6‐methyl and 7‐methyl brGDGT isomers (e.g., IIa′/IIa_6_ and IIa_7_) that are widely abundant in natural settings, pointing to other microbial producers and/or hitherto untested culture conditions.

Lastly, the relationship between brGDGTs and pH in *S. usitatus* was significantly less pronounced than the relationship with temperature and was opposite to the cyclization pattern typically observed in environmental samples (Figure 2c,f, Figures S9 and S11). A similar lack of pronounced pH trends was previously observed in lacustrine microcosm experiments (Martínez‐Sosa et al., 2020) and molecular dynamics simulations (Naafs et al., 2021). The fact that the near‐universal environmental pH dependence of brGDGT cyclization number was absent or opposite in *S. usitatus* runs counter to the hypothesis that cyclization number has a direct physiological connection to pH (Raberg et al., 2022a; Weijers et al., 2007). Instead, our results tentatively support the hypothesis that cyclization number is linked to pH via changes in bacterial community composition (De Jonge et al., 2019, 2021; Naafs et al., 2021). In contrast to its limited effect on brGDGT cyclization, pH in *S. usitatus* cultures did significantly modulate brGDGT methylation at lower temperatures. The observed decrease in MBT′_5Me_ values in response to increased pH is consistent with the effects of soil pH on brGDGT methylation recently observed in subarctic soils (Halffman et al., 2022). We note, however, that the absence of 6‐ and 7‐methyl isomers and near absence of doubly cyclized brGDGTs in *S. usitatus* limits our ability to draw direct comparisons with environmental samples through other pH‐related indices.

### 4.3 Influence of oxygen limitation on brGDGT production

A temperature‐independent methylation response was observed in the brGDGT composition of *S. usitatus* when oxygen was limited to 5% and 1% O_2_. At both low oxygen concentrations, brGDGT IIa increased relative to that of the atmospheric (21% O_2_) experiment while brGDGT IIIa decreased (Figure 3a). The low overall degree of methylation in *S. usitatus* at the temperature of these experiments (25°C) led to only small changes in the MBT′_5Me_ index and makes it difficult to ascertain whether this is a systematic membrane homeostasis response to oxygen limitation. In addition, the presently unknown exact structure and functional properties of the uncommon brGDGTs IIIa‐2 and IIIb‐2 further complicate interpretations with respect to membrane homeostasis. However, the production of brGDGTIIIa‐2 and brGDGTIIIb‐2 in *S. usitatus* under oxygen limitation suggests these compounds have some potential as indicators of low oxygen in environmental settings (Figure 3b). Both brGDGTIIIa‐2 and brGDGTIIIb‐2 were only detected above trace levels in *S. usitatus* at 5% and 1% O_2_ conditions, whereas all other pH and temperature conditions tested at 21% O_2_ yielded trace quantities (<0.3%) or were below the detection limit. Determining whether brGDGT IIIa‐2 and brGDGT IIIb‐2 are environmentally relevant is an important step for assessing their potential value as a sedimentary oxygen proxy. Additionally, the overall response of environmental brGDGT distributions to oxygen limitation must be resolved before brGDGT IIIa‐2 and brGDGT IIIb‐2 can be applied to corrective measures or new approaches in brGDGT‐based proxies.

Uncovering the role of oxygen limitation on brGDGT producers *at large* is paramount to ensuring the accuracy of climate records based on these compounds. Numerous environmental studies have highlighted that brGDGT distributions can respond to variations in environmental redox state and dissolved oxygen (e.g., Loomis et al., 2011; Martínez‐Sosa & Tierney, 2019; van Bree et al., 2020; Weber et al., 2018; Wu et al., 2021; Yao et al., 2020), but no clear consensus has emerged yet on how to capture and account for oxygen effects. Culture‐based insights on brGDGT response to suboxic settings are limited, but in the case of *E. aggregans*, 1% O_2_ was tied to the synthesis of brGDGT Ia, the only conventional brGDGT identified in this organism (Halamka et al., 2021; Sinninghe Damsté et al., 2011). The increase in tetra‐methylated brGDGTs in *E. aggregans* in response to oxygen limitation conflicts with the increase in penta‐methylated brGDGTs in *S. usitatus* under similar oxygen restrictions. A suboxic increase in brGDGT Ia would correspond to a warmer‐than‐actual temperature signature using conventional brGDGT paleotemperature indices, whereas an increase in penta‐ or hexa‐methylated compounds would correspond to colder‐than‐actual temperature signal. While the enzymatic capacity of *E. aggregans* to produce penta‐ and hexa‐methylated brGDGTs remains unclear, the seemingly opposing trends in methylation number response to oxygen limitation in culture are consistent with the complicated relationship of brGDGTs with O_2_ observed in environmental samples and serves as an important example of the need to further investigate the role of oxygen in the brGDGT‐producing bacterial community.

### 4.4 Implications for brGDGT Biosynthesis

**FIGURE 4:**
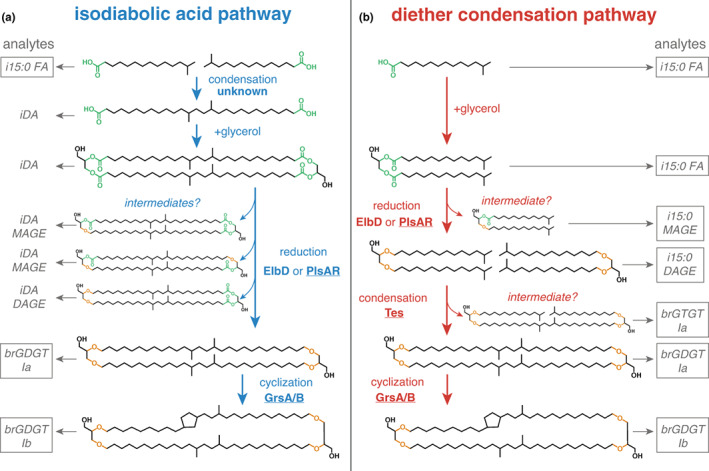
Hypothesized biosynthetic pathways for brGDGT production (excluding C5/C6 methylations). (a) (in blue): Pathway based on tail‐to‐tail condensation of two iso‐C15:0 FAs to form iso‐diabolic acid as a key intermediate in brGDGT biosynthesis, first proposed by Sinninghe Damsté et al. (2011, 2014, 2018). (b) (in red): Diether condensation pathway (Weijers et al., 2006) proposed for *Solibacter usitatus* based on the abundance of several potential intermediates and the existence of *S. usitatus* homologs of enzymes that perform similar functions in archaeal GDGTs and ester bond reduction in bacteria: Tes (tetra ether synthase), GrsA/B (GDGT ring synthesis), and PlsAR (plasmalogen synthase). Expected analytes produced by standard acid hydrolysis (which cleaves ester bonds, shown in green) for each pathway are listed on the far left and right side. Analytes that are boxed have been found in *S. usitatus*. Ester bonds shown in green; ether bonds shown in orange. Analytes: i15:0 FA = iso‐C15:0 fatty acid; iDA = iso‐diabolic acid; iDA MAGE = 1‐iso‐diabolic acid monoalkanoic glycerol monoether; iDA DAGE = 1,2‐iso‐diabolic acid dialkanoic glycerol diether; i15:0 MAGE = 1‐iso‐C15:0 monoalkyl glycerol monoether; i15:0 DAGE = 1,2‐iso‐C15:0 dialkyl glycerol diether; brGTGT Ia = branched glycerol trialkyl glycerol tetraether

Several lines of evidence suggest that *S. usitatus* may have a different pathway for brGDGT biosynthesis than what has been proposed for other Acidobacteria. Most Acidobacteria, including those previously discovered to synthesize brGDGT Ia, produce the membrane‐spanning iso‐diabolic acid (iDA in Figure 4) as a major membrane component (Sinninghe Damsté et al., 2011). In addition, several Acidobacteria produce a monoether of iso‐diabolic acid (iDA MAGE in Figure 4), which is particularly prominent in acid hydrolysis extracts from SD 4 Acidobacteria and occurs with additional methylations at the C5 position (Sinninghe Damsté et al., 2014). Based on the abundance of these likely brGDGT precursors and the known possibility of tail‐to‐tail condensation of FAs to make other membrane‐spanning di‐acids (Fitz & Arigoni, 1992), Sinninghe Damsté et al. (2011, 2014) proposed iso‐diabolic acid synthesis via the condensation of two iso‐C15 FAs by a still unknown enzyme as the first step toward brGDGT synthesis (Figure 4a, iso‐diabolic acid pathway). The discovery of an operon for bacterial ether lipid biosynthesis (elb) in myxobacteria (Lorenzen et al., 2014) then provided a potential mechanism for the conversion of ester to ether bonds to form mono‐, di‐, and eventually tetraethers by the ElbD enzyme as the second key step of this proposed pathway (Figure 4a), with several SD 4 genomes containing homologs of the entire elb operon (Sinninghe Damsté et al., 2018).

Contrary to most Acidobacteria studied to date, *S. usitatus* does not have detectable levels of iso‐diabolic acid in its cellular membrane (Table S4 and Sinninghe Damsté et al., 2018), but the organism does produce iso‐C15 mono and diethers (i15:0 MAGE and i15:0 DAGE in Figure 4; Table S4 and Sinninghe Damsté et al., 2018), which have been found in several other Acidobacteria as well (Sinninghe Damsté et al., 2018). In addition, our results show that *S. usitatus* produces brGTGT equivalents of several brGDGTs. Based on these findings and the presence of several homologs of recently discovered enzymes involved in ether lipid biosynthesis in bacteria and archaea (Jackson et al., 2021; Zeng et al., 2019, 2022), we propose an alternative pathway for brGDGT synthesis in *S. usitatus* (Figure 4b) based on tail‐to‐tail condensation of two iso‐C15 diethers akin to the biosynthesis of isoprenoidal GDGTs in Archaea (Galliker et al., 1998; Nemoto et al., 2003; Zeng et al., 2022) and as originally suggested for bacterial tetraether synthesis by Weijers et al. (2006). We hypothesize that in *S. usitatus*, the conversion of ester to ether lipids is the first step toward brGDGT synthesis and involves homologs of the plasmalogen ether lipid synthase PlsAR (Table S5, Figure S10) instead of ElbD, which *S. usitatus* lacks (Figure S10). PlsAR was discovered to mediate the reduction in ester to ether bonds in the anaerobic bacterial pathogen *Clostridium perfringens* (Jackson et al., 2021) and has been proposed to perform a similar function in the synthesis of ether lipids in the bacterium *Thermotoga maritima* (Sahonero‐Canavesi et al., 2022). Next, we suggest that the condensation of the resulting iso‐C15 diethers is mediated by one or both *S. usitatus* homologs of the tetraether synthase Tes enzyme (Figure S10), which is involved in the synthesis of isoprenoidal GDGTs from diether precursors in archaea and also produces GTGTs as potential intermediates (Zeng et al., 2022). Lastly, we suggest that the homologs of the archaeal GDGT ring synthases GrsA and GrsB in *S. usitatus* (Figure S10) could be involved in the formation of pentacyclic brGDGTs akin to their function in the formation of cyclized isoprenoidal GDGTs in archaea (Zeng et al., 2019).

Although we propose the above diether condensation pathway for *S. usitatus* (Figure 4b) based on potential intermediates and recent enzyme discoveries, it is possible that an iso‐diabolic acid pathway exists instead or in addition in this organism. Intermediates in biosynthetic pathways only accumulate at rate‐limiting steps and the findings reported in Halamka et al. (2021) suggest that the abundance of iso‐diabolic acid in the membrane of the SD1 Acidobacterium *E. aggregans* decreases with increased brGDGT production. The apparent absence of iso‐diabolic acid in *S. usitatus* thus cannot rule out its potential role in brGDGT synthesis at a step that is not rate‐limiting, leading to a subsequent lack of measurable iso‐diabolic acid in this organism. Future work using isotopic tracers in vivo and/or purified enzyme fractions in vitro has the potential to resolve the exact pathway of brGDGT biosynthesis in *S. usitatus*.

## 5 FUTURE DIRECTIONS

This study demonstrates that the degree of brGDGT methylation in a single bacterial species functions as a physiological response to changing temperature, with distributions that are in strong agreement with environmental observations. The results serve as laboratory‐based support for the use of brGDGTs as a paleothermometer, while also presenting possible caveats for the effects of pH and O_2_ on brGDGT proxies as well as new potential opportunities for using some brGDGT structures to identify suboxic conditions in the past. The results of this study do not demonstrate a clear relationship between the degree of brGDGT cyclization and pH. Instead, our findings underscore the need for further investigation into the effects of microbial community structure as well as other potential physiological factors such as nutrient availability and carbon sources on brGDGT cyclization. Detection of brGDGTs with varying degrees of cyclization and methylation suggest that *S. usitatus* can serve as a potential genetic system to test hypotheses about the biosynthesis of brGDGTs in culture, as well as studies on the evolutionary origin of genes involved in brGDGT synthesis in bacteria.

## CONFLICT OF INTEREST

The authors declare no competing interests.

## Supporting information


Appendix S1

Appendix S2

Appendix S3

## Data Availability

The data that support the findings of this study are available in the supplementary material of this article. All data from this study were processed in R (R Core Team, 2021) and are available in Tables S1–S5 as well as in spreadsheet format in Datasets S1 and S2. All code is available at https://github.com/KopfLab/2022_halamka_et_al.
