# Supplementary material for: Production of diverse brGDGTs by Acidobacterium Solibacter usitatus in response to temperature, pH, and O2 provides a culturing perspective on brGDGT proxies and biosynthesis
Source: Geobiology. 2022 Sep 23;21(1):102–18. doi: 10.1111/gbi.12525 (PMC10087280; doi:10.1111/gbi.12525)
Supplement: Supplementary file 3 — Appendix S3 [file GBI-21-102-s001.pdf]

Supplementary Information for

Production of diverse brGDGTs by Acidobacterium *Solibacter usitatus* in response to temperature, pH, and O_2 provides culturing perspectives on brGDGT proxies and biosynthesis

Toby A. Halamka[1*], Jonathan H. Raberg[1,2], Jamie M. McFarlin[1], Adam D. Younkin[1], Christopher Mulligan[1], Xiao-Lei Liu[3], Sebastian H. Kopf[1]

[1]University of Colorado Boulder, Department of Geological Sciences
[2]University of Iceland, Faculty of Earth Sciences
[3]University of Oklahoma, School of Geosciences

Corresponding author: *Toby A. Halamka
Email: Toby.Halamka@colorado.edu

This PDF file includes:

- Figures S1 to S13
- Tables S1 to S7
- SI References

Other supplementary materials for this manuscript include the following:

- Dataset S1: Excel spreadsheet with growth rate and lipid data
- Dataset S2: Excel spreadsheet with environmental and culture climate proxy data and regression fits

Supplementary Figures

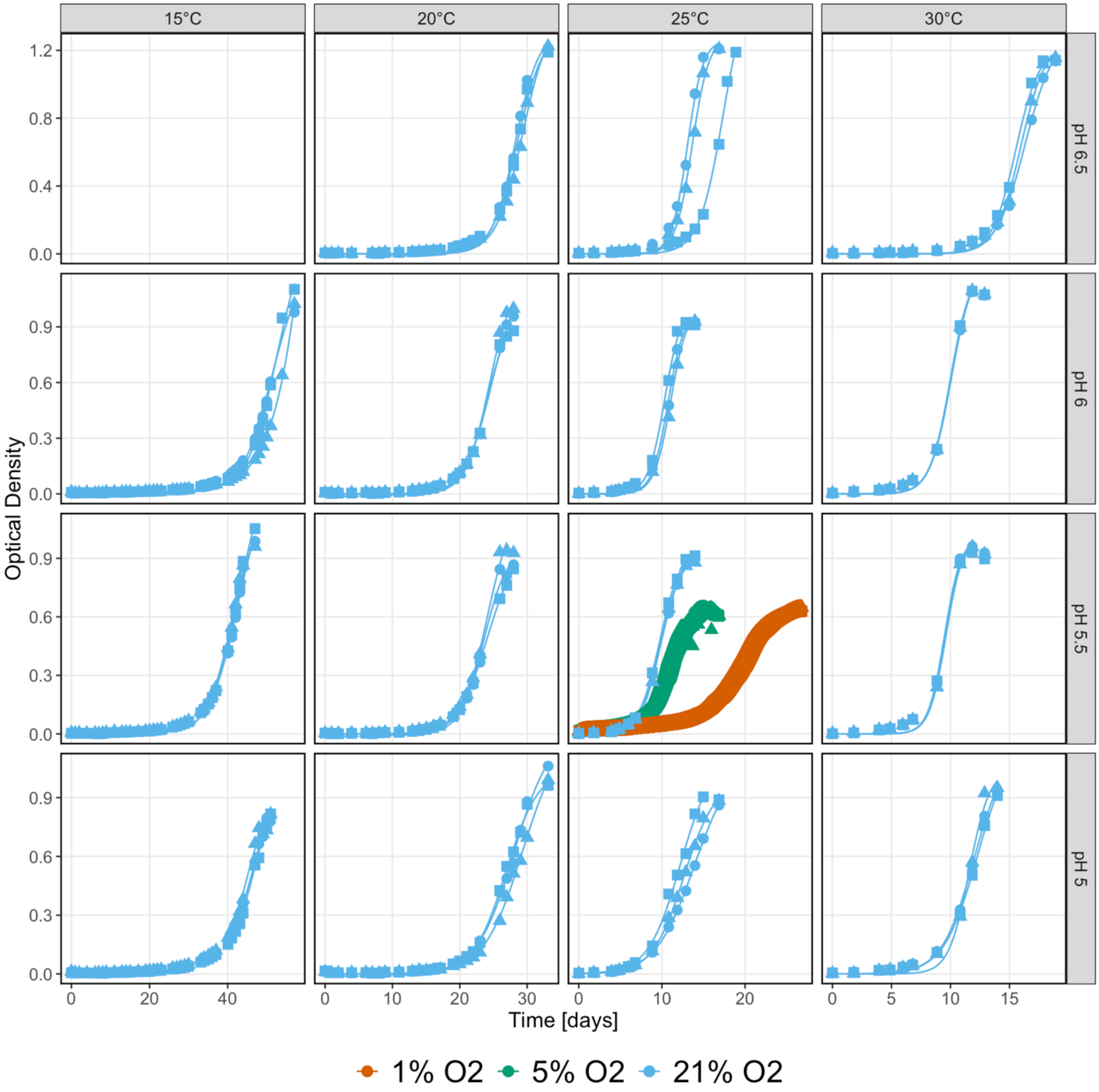

Fig. S1. Growth curves of *S. usitatus* at different oxygen concentrations, temperatures and pH. Symbol shapes differentiate replicate cultures. Lines are fits to the logistic equation for growth rate estimates (see Fig. S3 and Table S1 for details). Optical density measurements for suboxic experiments were recorded automatically as described in the Materials & Methods and thus have much higher data density than manually recorded growth curves. For

visual clarity, optical density measurements for suboxic experiments are rescaled in this figure (1 : 3.96) to adjust for the longer pathlength through the 100mL bottles and thus 3.96x higher OD readings.

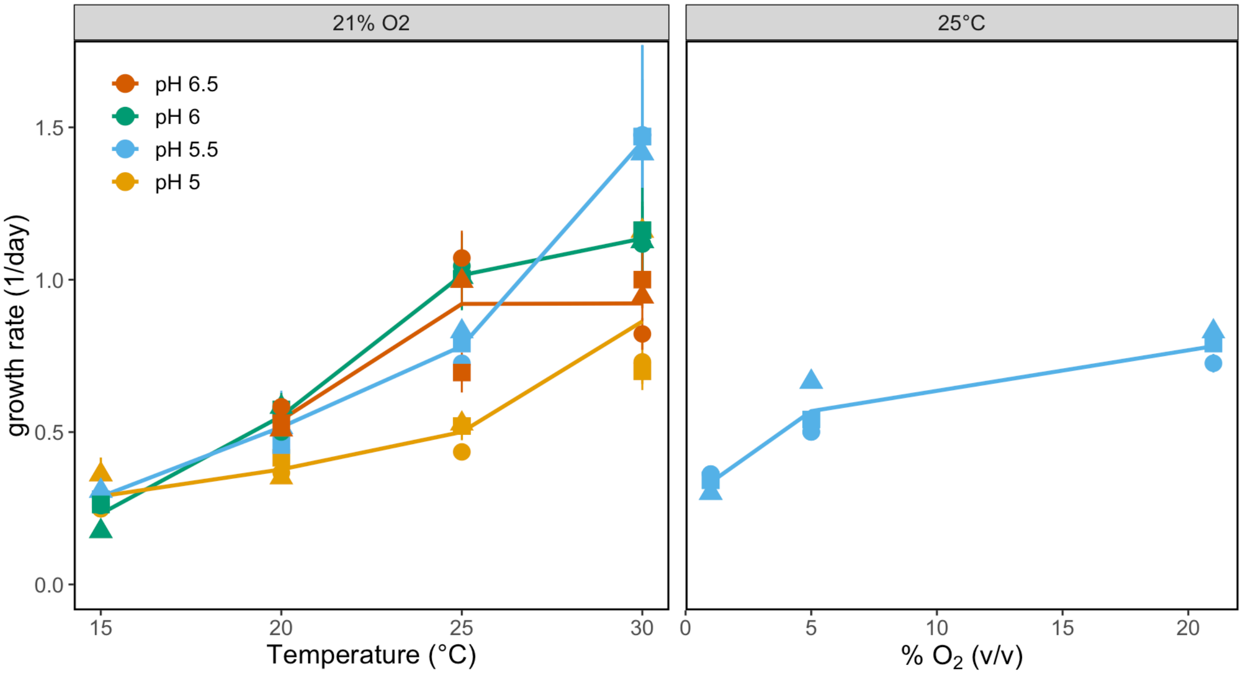

Fig. S2. Growth rates of *S. usitatus* at different oxygen concentrations, temperatures and pH. Shapes differentiate replicate cultures. Error bars indicate standard errors of growth rate estimates from regression fits. Some error bars are smaller than symbol sizes. See Table S1 for all numerical values.

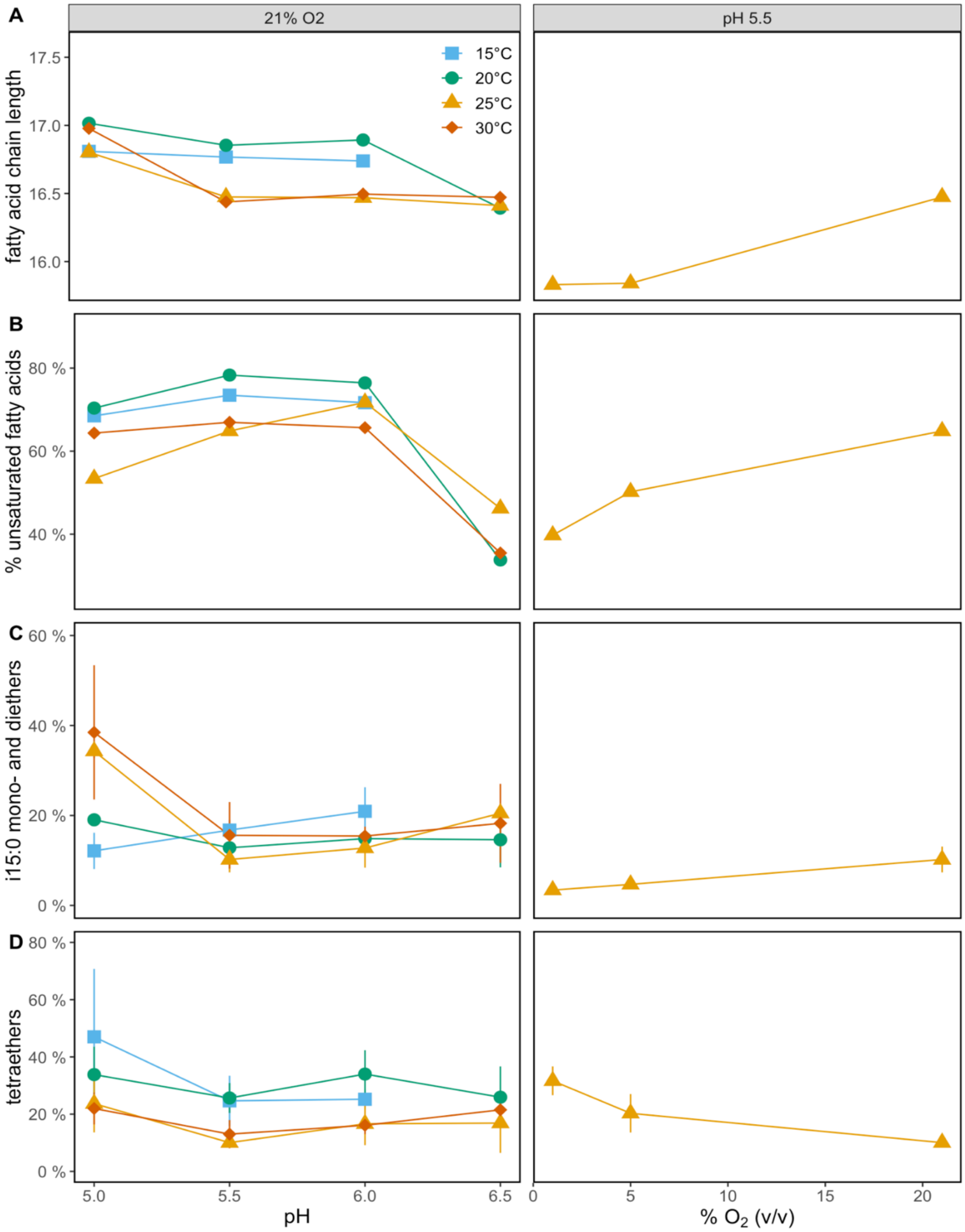

Fig. S3. Lipid abundance patterns of *S. usitatus* across all experiments vs pH at 21% O_2 on the left and vs. %O_2 at pH 5.5 on the right. A: weighted average carbon chain length of all fatty acids. B: weighted average unsaturation of all fatty acids. C: overall abundance of mono and diethers. D: overall abundance of tetraethers. See Tables S1-S4 for underlying data.

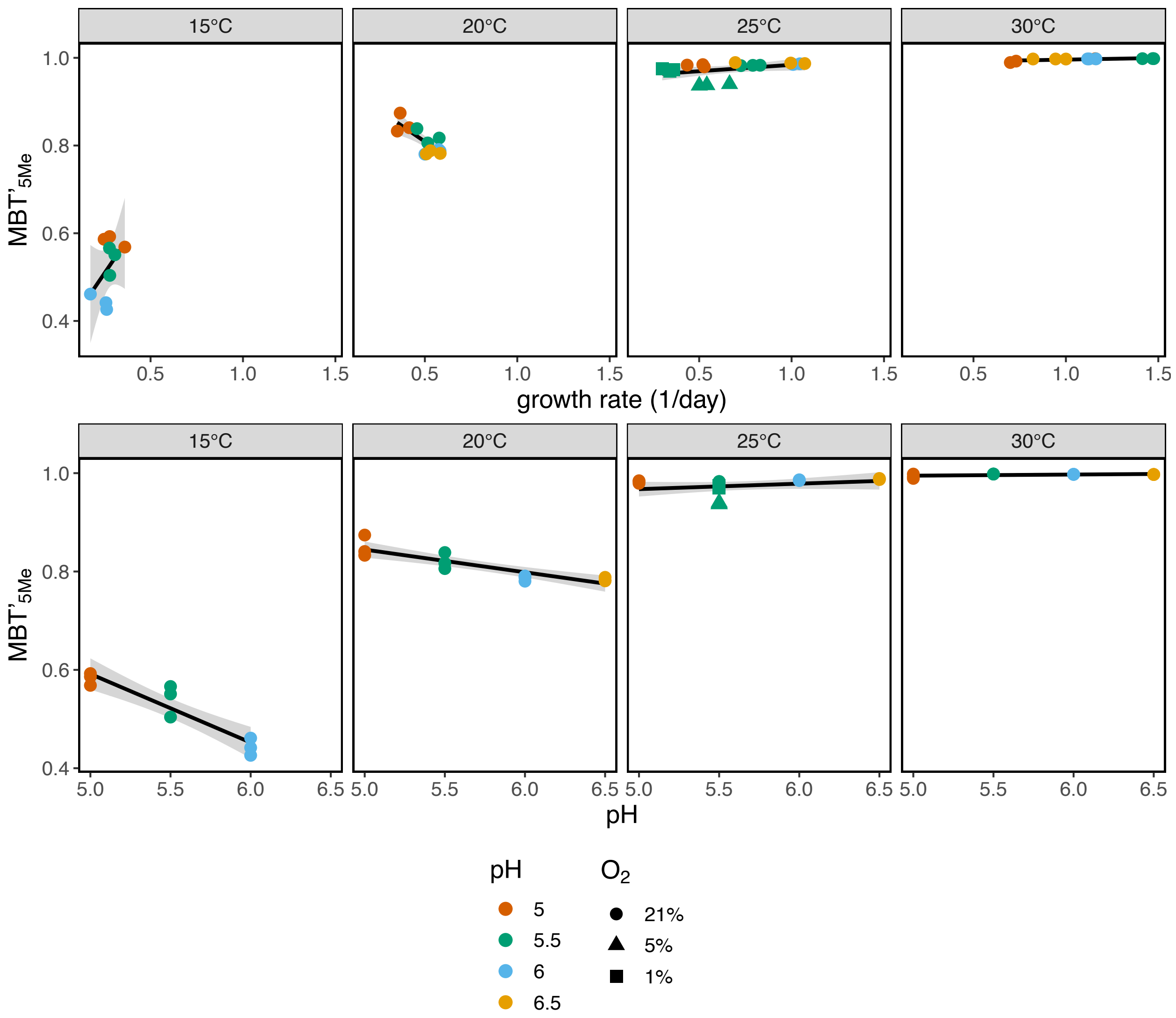

Fig. S4. MBT'$_{5Me}$ index vs. growth rate (top) and vs. pH (bottom). Symbols represent oxygen concentrations. Colors represent pH values for visual clarity. Linear regression lines are shown with 95% confidence intervals (see Table 1 in the main text for correlation and regression coefficients). All culture data is shown. Biological replicates plot on top of each other in some cases. See Tables S1 and S5 for underlying data.

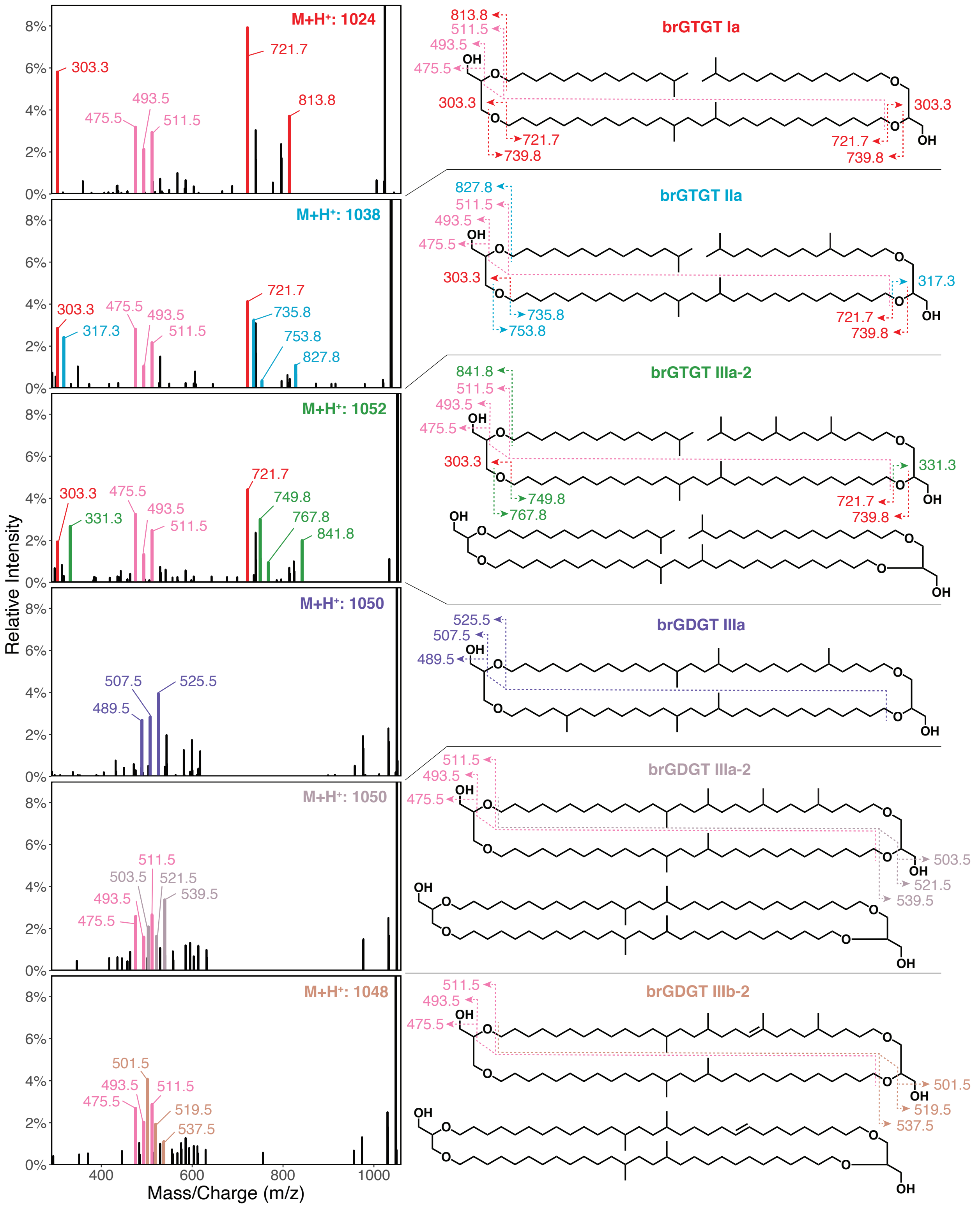

Fig. S5. MS/MS spectra, fragmentation patterns and proposed structures of brGTGTs and uncommon brGDGTs observed in *S. usitatus*. Left: MS/MS spectra, intensities are scaled to molecular ion ($M+H^+$ peak)

in each panel. Right: common fragments across tetraethers are highlighted in the same colors (pink & red) while characteristic fragments of each tetraether are highlighted in their own color (brGDGTs are color-coded as in Fig. 1 and Fig. 3). Both proposed structures for brGTGT IIIa-2, brGDGT IIIa-2 and brGDGT IIIb-2 are consistent with the respective fragmentation pattern. The exact location of the methyl branches for overly branched structures is speculative (but must be on the same alkyl chain in brGTGT IIIa-2). The double bond in brGDGT IIIb-2 could be a ring instead (structure not shown).

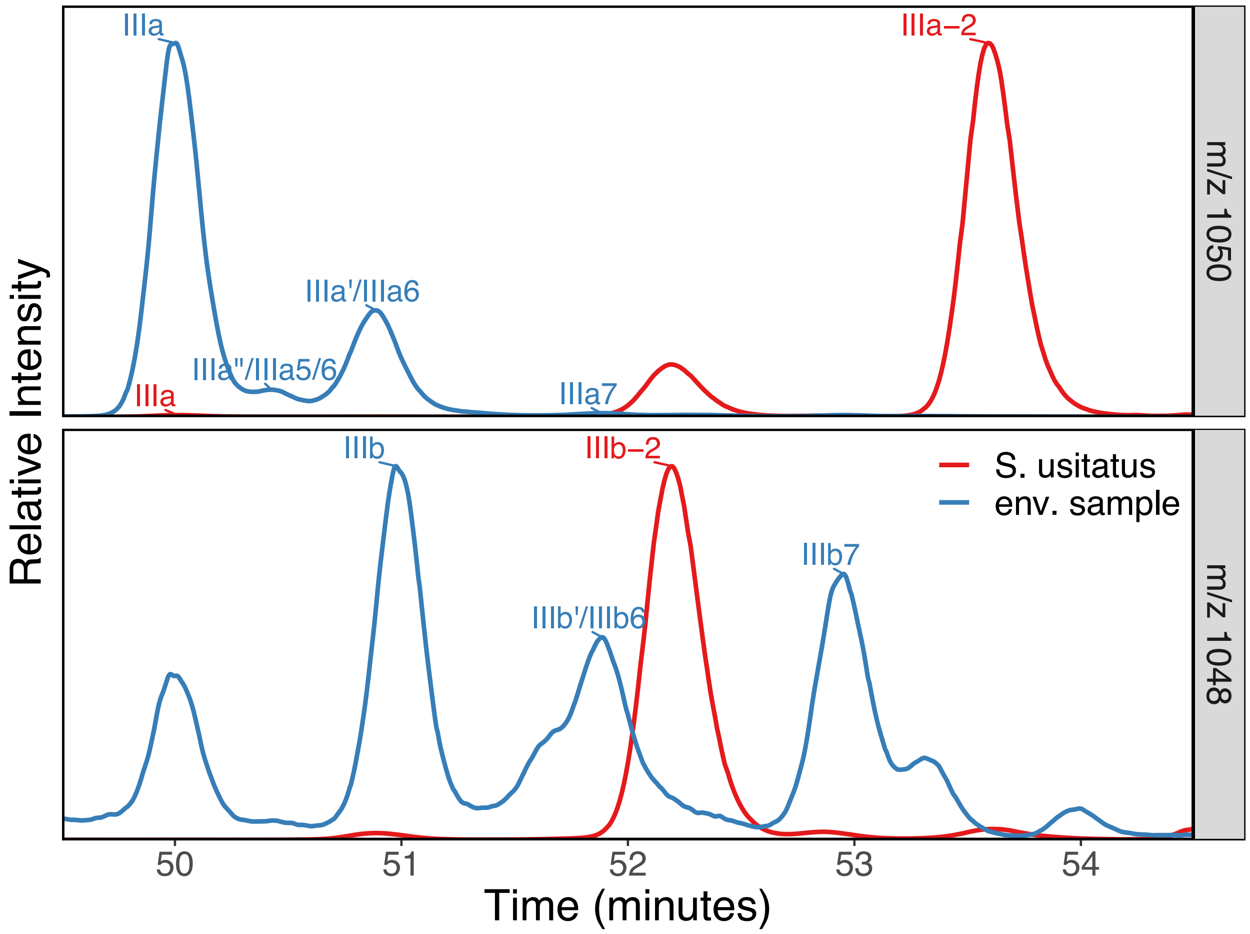

Fig. S6. NP-HPLC Mass Extracted Chromatograms of brGDGT IIIa isomers (m/z 1050) and brGDGT IIIb isomers (m/z 1048) in a 1% O2 culture (red line) of *S. usitatus* and the environmental reference sample (blue line). Peak intensities are normalized to the largest peak in the shown time window for each mass extracted chromatogram. Relative elution times are highlighted with red arrows. BrGDGT IIIa (50.0 min), IIIa-2 (53.6 min) and IIIb-2 (52.2 min) are color-coded as in Fig. 1, Fig. 3, Fig. S5. (50.4), IIIa'/IIIa$_6$ (50.9), IIIa$_7$ (51.9), IIIb (50.9), IIIb'/IIIb$_6$ (51.9) and IIIb$_7$ (53.0) in black are not found in *S. usitatus*.

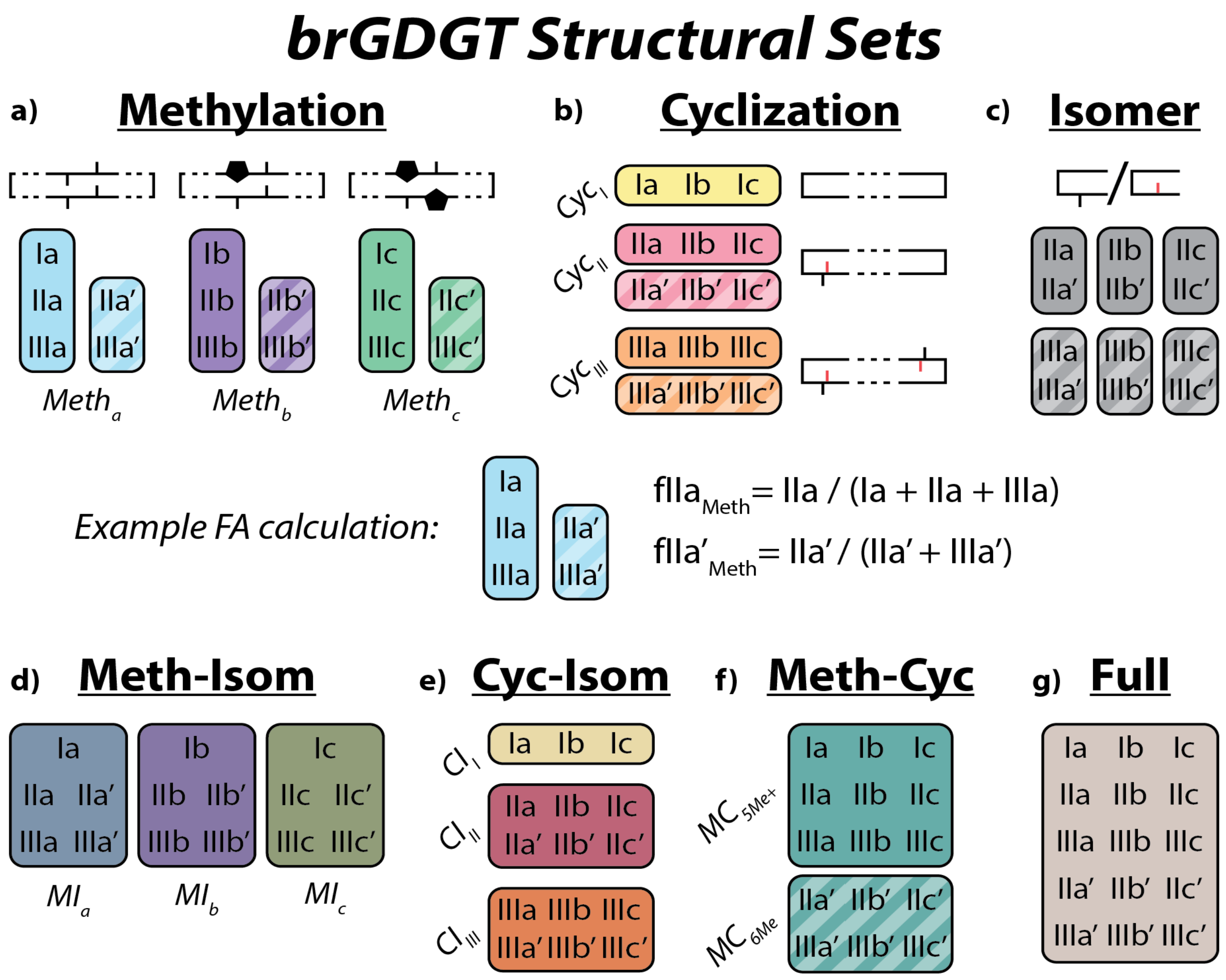

Fig. S7. Schematic of the basic (a-c) and combined (d-g) brGDGT structural sets. Fractional abundances are calculated within each boxed group independently (example calculation in center). Schematic structures highlight the defining alkyl-chain moieties, with cyclopentane rings filled in for emphasis and C6 methylations denoted in red. Reproduced from Raberg et al. (2021).

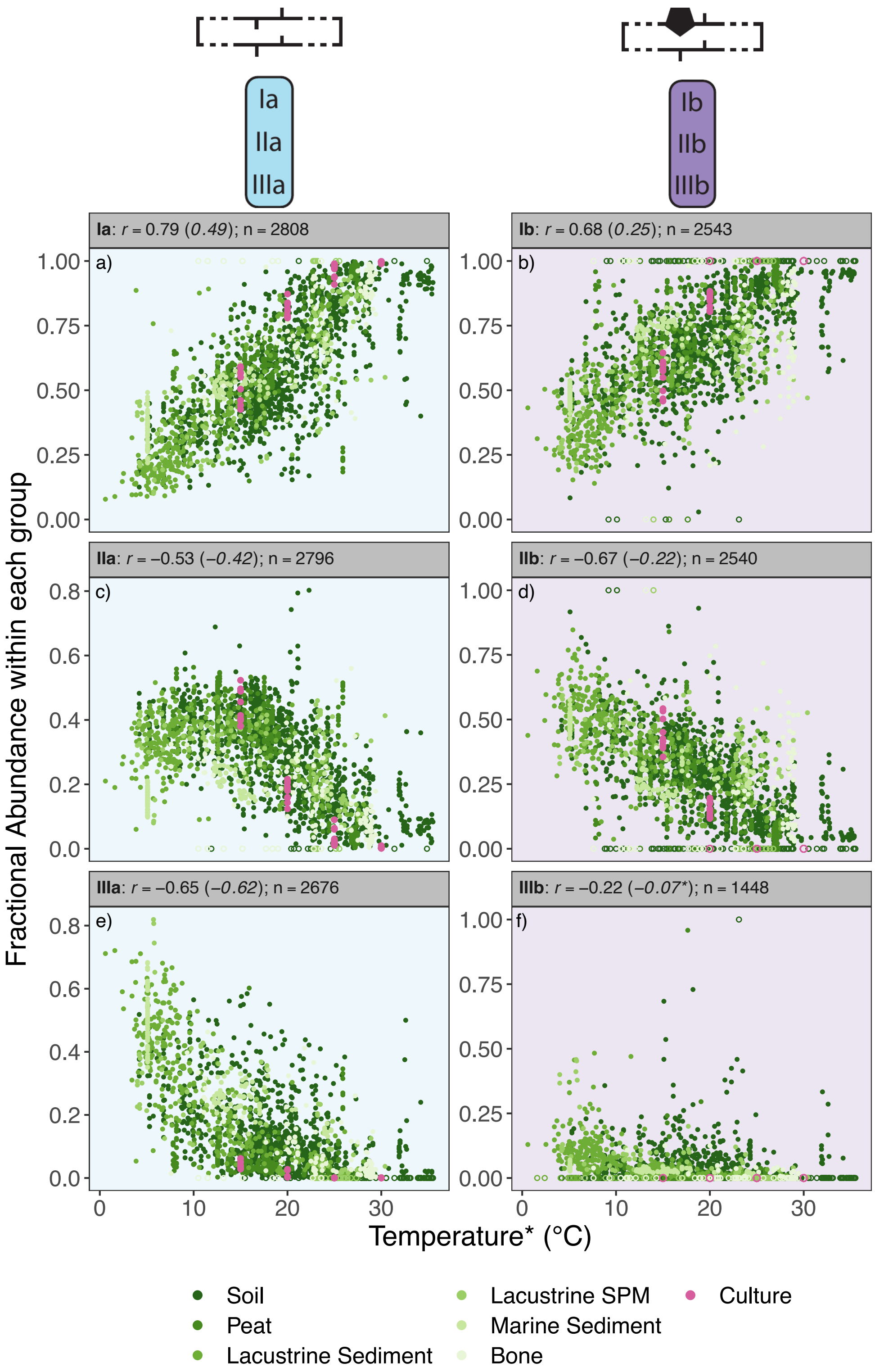

Fig. S8. Relationship between Methylation Set fractional abundances (FAs) of 5-methyl acyclic and monocyclic brGDGTs and temperature. Linear correlation coefficients *r* across *n* samples are provided for each subplot, with coefficients for the standard Full FAs given in parentheses for comparison. P values

were <0.01 except where marked with an asterisk. Samples with FA = 0 or 1 were treated as outliers and removed from statistical analyses (*r*, *n,* and p values). *Temperatures were associated with different sample types following Raberg et al. (2022a). Schematics of Methylation Set groupings are provided at top.

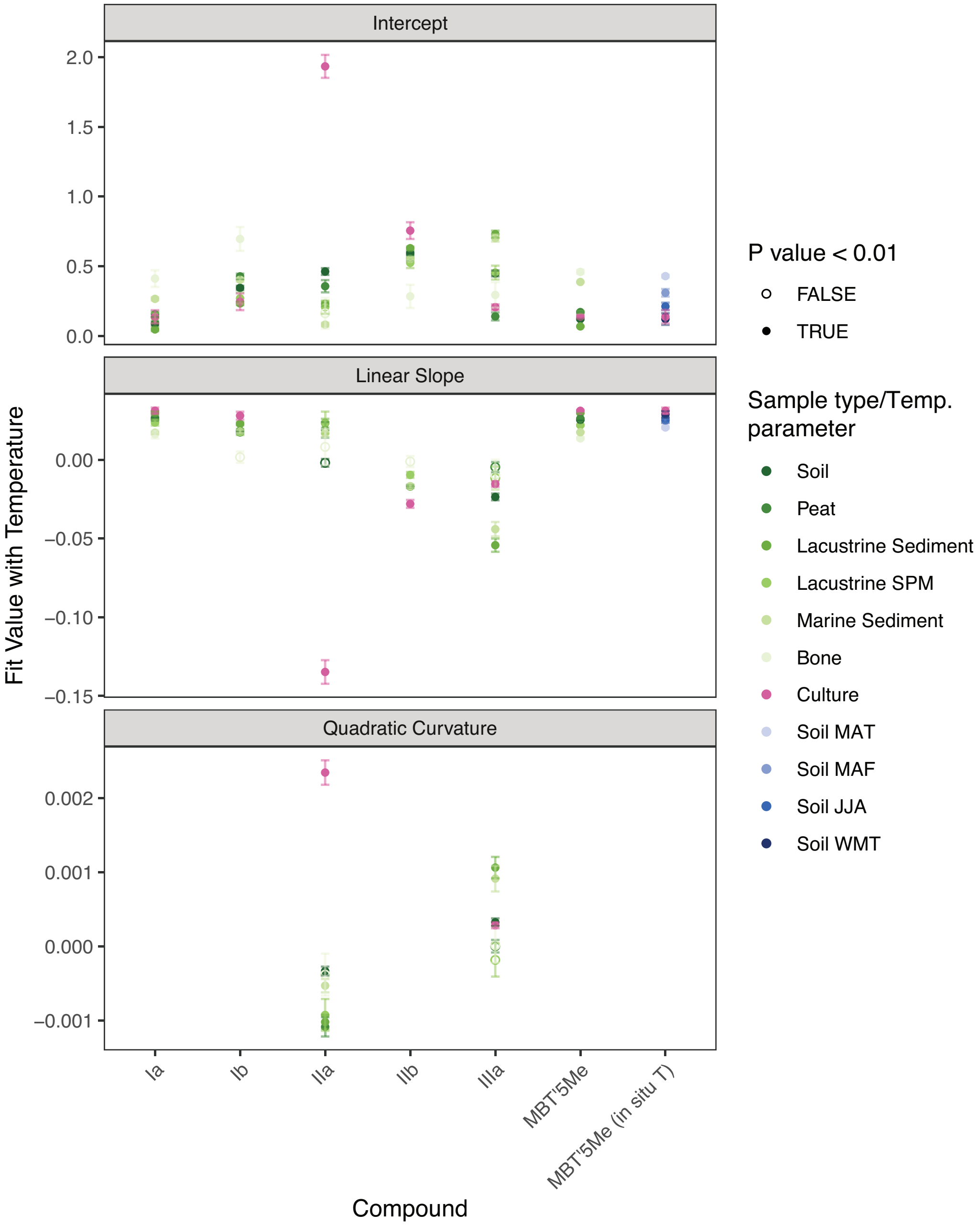

Fig. S9. Fitting coefficients for quadratic (**II**a and **III**a) and linear (all others) regressions between brGDGT Methylation Set fractional abundances, MBT'$_{5Me}$ and temperature, as plotted in Figures S8 and 2D, as well as between MBT'$_{5Me}$ and *in situ* temperature as plotted in Figure 2E. Error bars represent one standard

error. Coefficients with p values ≥ 0.01 are plotted as open circles. Abbreviations are defined in Figure 2 caption. See Table 1 for MBT'$_{5Me}$ regression parameters.

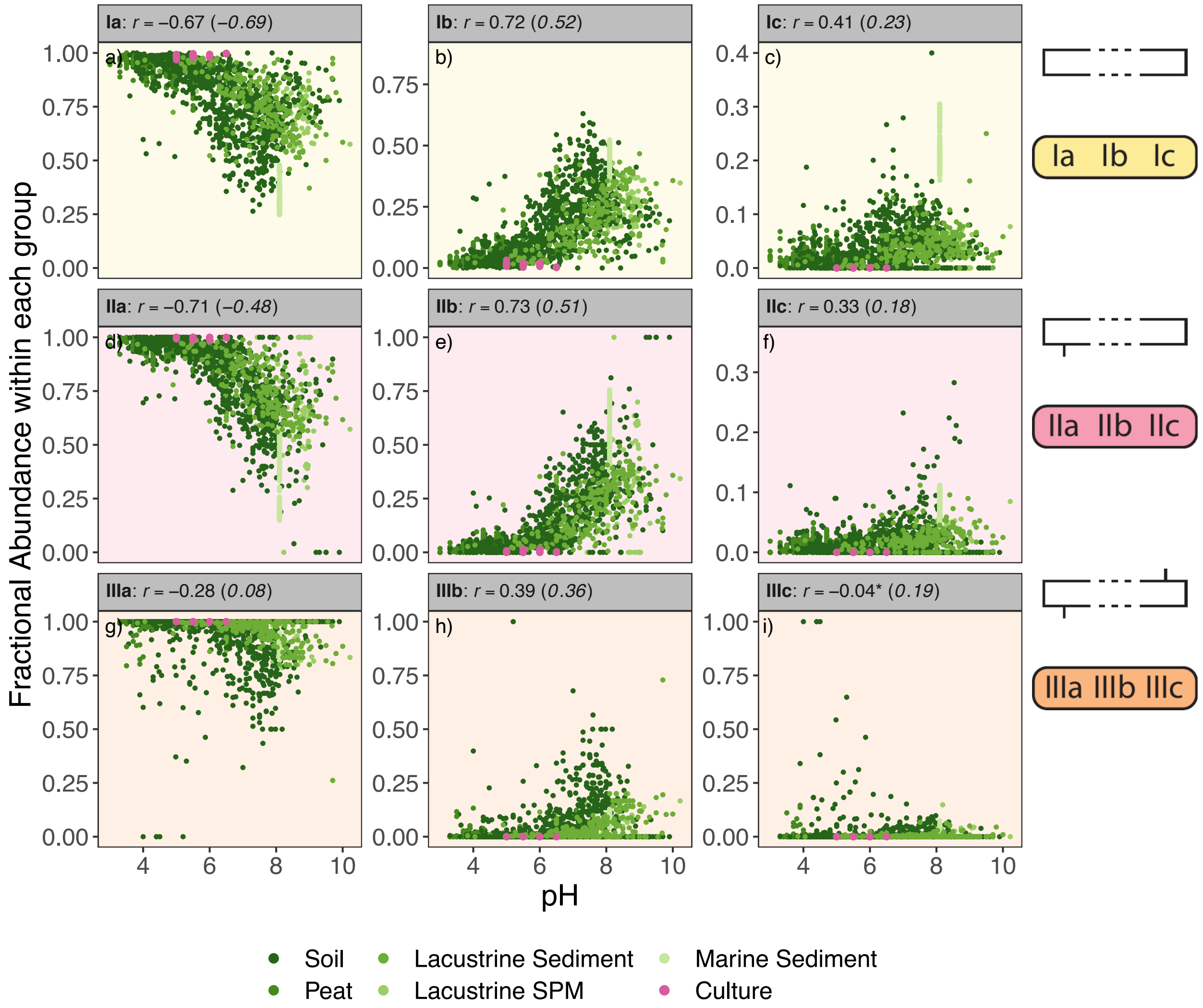

Fig. S10. Relationship between Cyclization Set fractional abundances (FAs) of 5-methyl brGDGTs and pH. Linear correlation coefficients *r* are provided for each subplot (*n* = 1856 for all), with coefficients for the standard Full FAs given in parentheses for comparison. P values were <0.01 except where marked with an asterisk. Schematics of Cyclization Set groupings are provided at right.

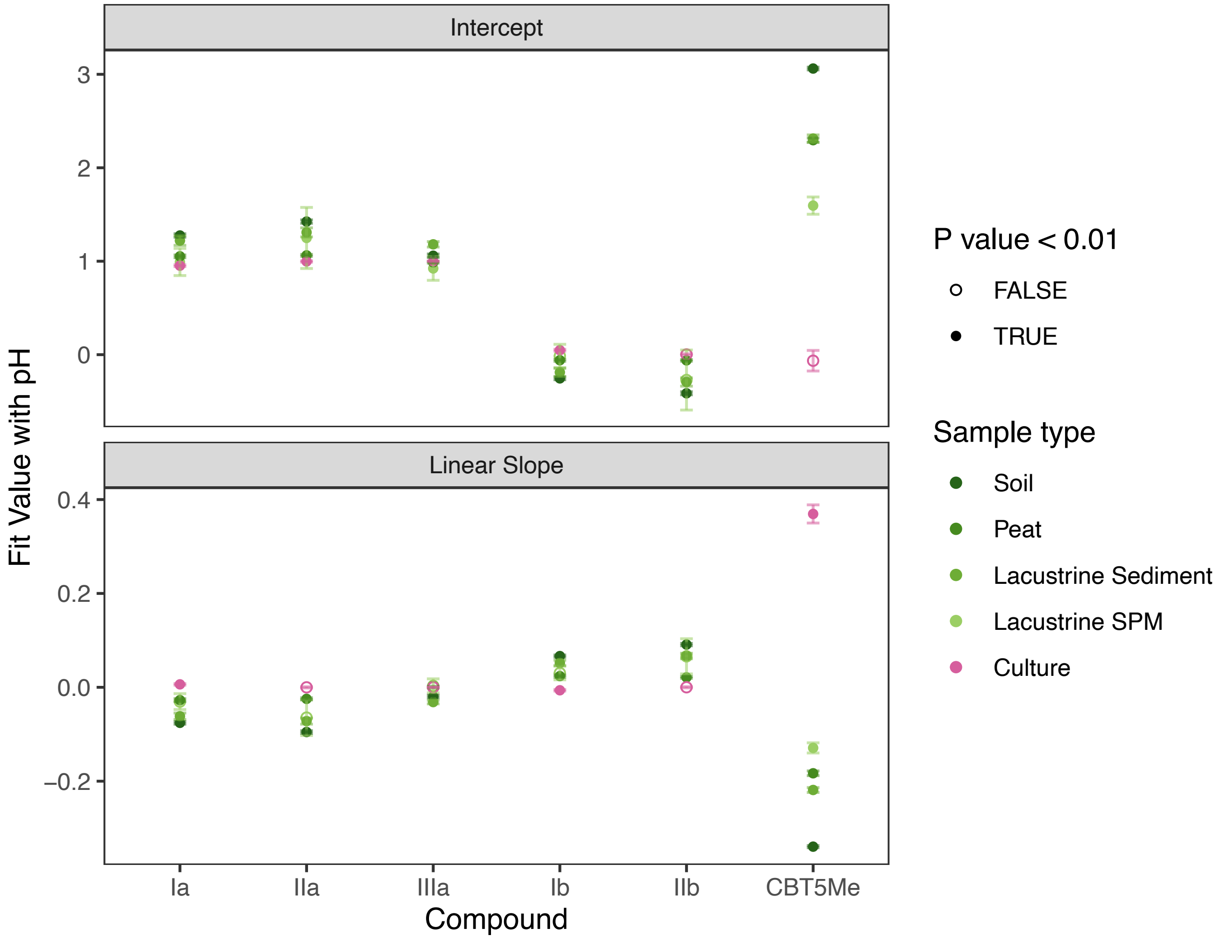

Fig. S11. Fitting coefficients for linear regressions between brGDGT Cyclization Set fractional abundances, CBT_{5Me}, and pH, as plotted in Figures S10 and 2F. Error bars represent one standard error. Coefficients with p values ≥ 0.01 are plotted as open circles. See Table 1 for CBT_{5Me} regression parameters.

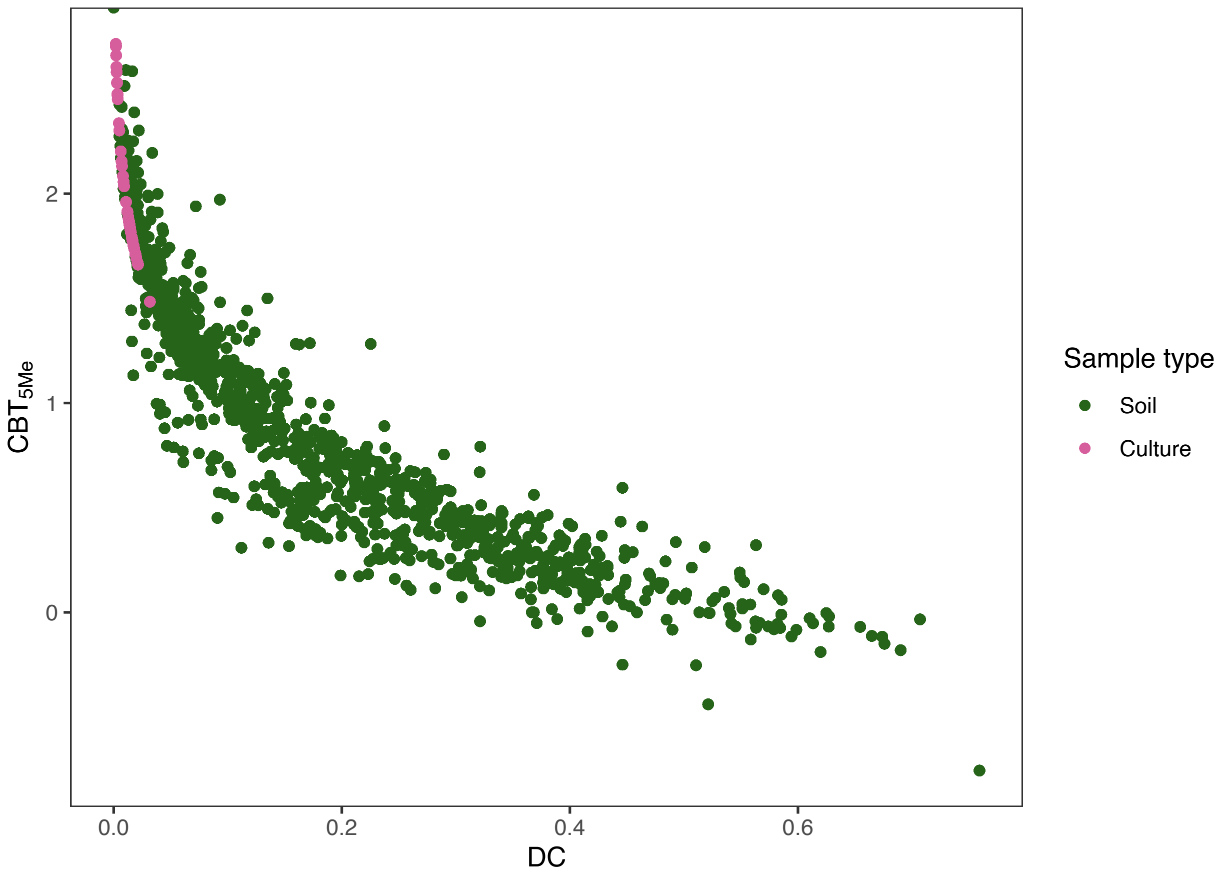

Fig. S12. Relationship between CBT_{5Me} and Degree of Cyclization (DC; Baxter et al., 2019), showing the high sensitivity of CBT_{5Me} when DC is low.

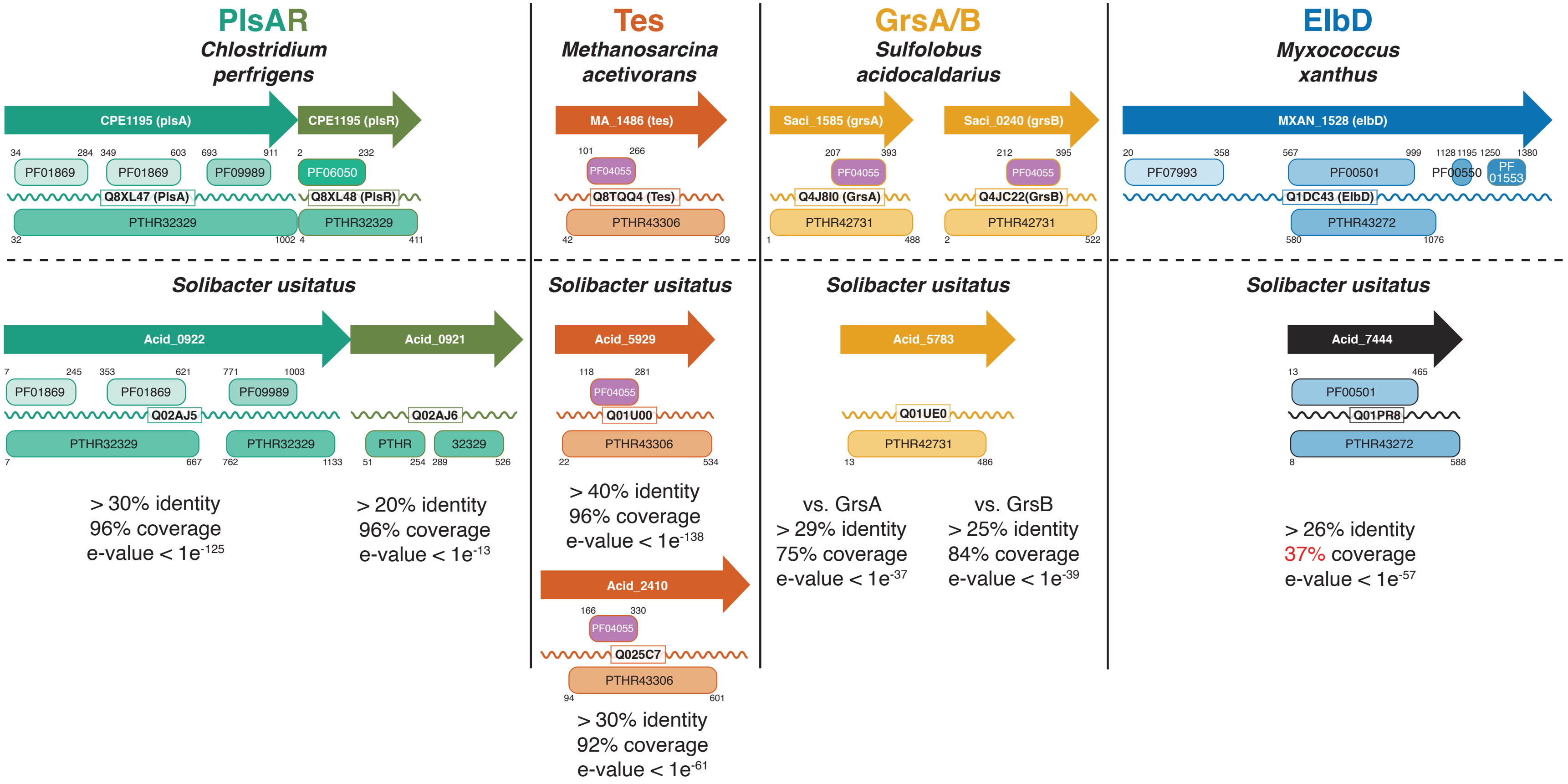

Fig. S13. Overview of *S. usitatus* homologs of proteins potentially involved in brGDGT biosynthesis. Genes are represented by arrows inscribed with gene loci. Proteins are represented by zigzag lines inscribed with UniProtIDs. Protein family and domain classifications (as predicted by Interpro scan, Hunter et al., 2009) are represented by rounded rectangles with PFAM (prefix "PF", Mistry et al., 2021) depicted above zigzag protein lines, and PANTHER (prefix "PTHR", Mi et al., 2013) depicted below zigzag protein lines. All representations are to scale with respect to amino acid sequence lengths. Proteins are color-coded for clarity except for the radical SAM domain (PF04055, in purple) which is part of both Tes and GrsA/B

proteins. Protein BLAST scores of *S. usitatus* homologs are provided below each protein (see Table S5 for details). PlsAR (ether lipid biosynthesis), Tes (tetraether synthesis) and GrsA/B (GDGT ring synthesis) from *Clostridium perfrigens*, *Methanosarcina acetivorans*, and *Sulfolobus acidocaldarius*, respectively, all have close homologs and domain structure in *S. usitatus*. ElbD (ether lipid biosynthesis) from *Myxococcus xanthus* and one of its BLAST results in *S. usitatus* is also included and shows how only the PF500501 portion of the protein (AMP-binding domain) matches proteins in *S. usitatus* (all with less than 40% coverage).

Supplementary Information Tables

Table S1. Culture growth rates (μ) and generation estimates for all experiments with *S. usitatus*. Growth rates were calculated for all replicates by fitting OD measurements to the logistic equation below using non-linear least squares regressions in R (t is time, fit parameters μ and K represent the growth rate and carrying capacity / max OD): $\boldsymbol{OD\ (t)} = \frac{K}{1+(K/OD_{t0}-1)\cdot e^{-\mu t}}$. Growth rates of individual replicates are listed with the standard errors of the regression fit. Growth rate averages are listed with the standard deviation of the replicates. Generation estimates were calculated based on optical densities of the inoculum and final optical densities of the cultures at harvest and are averaged across replicate cultures: $\#\boldsymbol{gen} = \log_2 \frac{OD_{tmax}}{OD_{t0}}$. See Fig. S1 for visualization of growth curves and Fig. S2 for visualization of growth rates. See Dataset S1 for these data in spreadsheet format.

Temperature (°C)	pH	% O_2 (v/v)	Growth rates (day^{-1})				
			Rep. 1	Rep. 2	Rep. 3	Average	#gen
15	5.0	21	0.25±0.02	0.36±0.06	0.28±0.03	0.30±0.06	8.02±0.02
15	5.5	21	0.28±0.02	0.31±0.03	0.28±0.02	0.29±0.02	8.32±0.07
15	6.0	21	0.26±0.03	0.17±0.01	0.26±0.03	0.23±0.05	8.37±0.09
20	5.0	21	0.37±0.02	0.35±0.01	0.42±0.02	0.38±0.03	8.65±0.07
20	5.5	21	0.58±0.06	0.52±0.05	0.46±0.01	0.52±0.06	8.47±0.08
20	6.0	21	0.50±0.03	0.58±0.05	0.57±0.04	0.55±0.05	8.56±0.09
20	6.5	21	0.58±0.04	0.51±0.03	0.53±0.04	0.54±0.04	8.92±0.02
25	5.0	21	0.43±0.01	0.53±0.02	0.52±0.05	0.49±0.05	8.21±0.03
25	5.5	21	0.73±0.03	0.83±0.03	0.79±0.02	0.78±0.05	8.24±0.03
25	6.0	21	1.04±0.11	1.01±0.10	1.00±0.10	1.02±0.02	8.28±0.00
25	6.5	21	1.07±0.09	1.00±0.08	0.70±0.07	0.92±0.20	8.66±0.01
30	5.0	21	0.73±0.07	1.16±0.21	0.70±0.06	0.86±0.26	8.29±0.03
30	5.5	21	1.48±0.29	1.41±0.24	1.47±0.30	1.45±0.03	8.32±0.03
30	6.0	21	1.12±0.12	1.13±0.13	1.16±0.14	1.14±0.02	8.52±0.00
30	6.5	21	0.82±0.05	0.94±0.08	1.00±0.09	0.92±0.09	8.59±0.01
25	5.5	5	0.50±0.00	0.66±0.00	0.54±0.00	0.57±0.09	6.37±0.03
25	5.5	1	0.36±0.00	0.30±0.00	0.34±0.00	0.33±0.03	6.36±0.02

Table S2. Overall membrane composition estimates for *S. usitatus* for all experimental conditions. Tetraether abundances were calculated relative to fatty acids and mono/di-ethers using the C24:0 fatty acid and C46 GTGT internal standards. Reported relative abundances are the statistical means and standard deviations of biological triplicates. The last column ('All') represents the statistical average and standard deviation across all experiments. See Dataset S1 for these data in spreadsheet format.

**Experimental Conditions**																		
**T (°C)**	**15**	**15**	**15**	**20**	**20**	**20**	**20**	**25**	**25**	**25**	**25**	**30**	**30**	**30**	**30**	**25**	**25**	**All**
**pH**	**5.0**	**5.5**	**6.0**	**5.0**	**5.5**	**6.0**	**6.5**	**5.0**	**5.5**	**6.0**	**6.5**	**5.0**	**5.5**	**6.0**	**6.5**	**5.5**	**5.5**	
**% O2**	**21**	**21**	**21**	**21**	**21**	**21**	**21**	**21**	**21**	**21**	**21**	**21**	**21**	**21**	**21**	**5**	**1**	
**Major lipid classes' relative abundances in % (mean ± 1 standard deviation)**																		
fatty acids	40.9 ±23.8	58.6 ±11.0	53.9 ±8.3	47.1 ±9.2	61.5 ±8.2	51.1 ±8.6	59.5 ±16.2	42.0 ±11.5	79.7 ±2.8	70.6 ±11.8	62.6 ±10.0	39.6 ±20.3	71.4 ±12.2	68.4 ±5.2	60.2 ±12.3	75.0 ±6.7	64.9 ±5.7	**59.2 ±12.1**
monoethers & diethers	12.1 ±4.0	16.8 ±2.2	20.9 ±5.4	19.0 ±0.9	12.8 ±3.3	14.9 ±1.0	14.6 ±6.1	34.3 ±1.5	10.2 ±2.9	12.8 ±4.4	20.5 ±2.0	38.5 ±14.9	15.6 ±7.4	15.4 ±2.1	18.3 ±8.8	4.7 ±0.3	3.4 ±0.7	**16.8 ±8.8**
tetraethers	47.0 ±23.7	24.7 ±8.8	25.2 ±3.4	33.8 ±9.7	25.7 ±5.2	34.0 ±8.3	25.9 ±10.7	23.7 ±10.1	10.1 ±0.1	16.6 ±7.4	16.9 ±10.4	22.0 ±5.6	13.0 ±4.9	16.1 ±3.1	21.5 ±4.8	20.3 ±6.7	31.6 ±5.0	**24.0 ±9.0**

Table S3. Gas chromatography data including fatty acids, mono-ethers and di-ethers for *S. usitatus* for all experimental conditions. Relative abundances for each sample were calculated from flame ionization detector (FID) peak areas (n.d. = not detected). Reported relative abundances are the statistical means and standard deviations of biological triplicates. Most unsaturated fatty acids (e.g., i15:1, i17:1, 18:1) were detected as multiple closely eluting isomers that reflect different positions of the double bond and were summed together for this data overview. The last column ('All') represents the statistical average and standard deviation across all experiments. See Table S7 for chemical structures and full names of key fatty acids and mono/diethers. See Dataset S1 for these data in spreadsheet format.

**Experimental Conditions**																	
**T (°C)**	**15**	**15**	**15**	**20**	**20**	**20**	**20**	**25**	**25**	**25**	**25**	**30**	**30**	**30**	**30**	**25**	**25**	**All**
**pH**	**5.0**	**5.5**	**6.0**	**5.0**	**5.5**	**6.0**	**6.5**	**5.0**	**5.5**	**6.0**	**6.5**	**5.0**	**5.5**	**6.0**	**6.5**	**5.5**	**5.5**	
**% O2**	**21**	**21**	**21**	**21**	**21**	**21**	**21**	**21**	**21**	**21**	**21**	**21**	**21**	**21**	**21**	**5**	**1**	
**Fatty acid relative abundances in % (mean ± 1 standard deviation)**																		
14:0	< 1	< 1	< 1	< 1	< 1	< 1	< 1	3.2 ±1.3	< 1	< 1	< 1	1.1 ±1.2	< 1	< 1	< 1	< 1	< 1	**< 1**
i15:1	1.6 ±1.8	1.2 ±1.3	< 1	< 1	3.7 ±1.7	1.6 ±0.8	1.7 ±2.2	< 1	5.4 ±2.8	4.6 ±3.0	1.6 ±0.4	< 1	4.0 ±3.6	2.2 ±1.5	< 1	9.8 ±0.3	3.4 ±0.1	**2.5 ±2.5**
i15:0	2.8 ±4.2	1.3 ±1.0	2.0 ±1.7	< 1	2.0 ±2.8	< 1	12.2 ±14.3	< 1	16.0 ±6.0	12.8 ±11.0	13.9 ±11.9	1.3 ±2.2	15.0 ±12.3	11.8 ±5.8	10.6 ±14.8	40.2 ±0.5	44.1 ±1.2	**11.0 ±13.2**
15:0	7.1 ±2.9	6.9 ±2.0	7.9 ±6.6	3.7 ±6.4	< 1	3.9 ±5.3	10.8 ±5.5	8.0 ±6.9	1.5 ±1.2	2.9 ±0.4	6.9 ±6.3	4.3 ±4.1	3.4 ±5.3	6.1 ±3.1	11.9 ±5.5	1.6 ±0.4	3.1 ±0.3	**5.3 ±3.2**
i16:0	< 1	< 1	< 1	< 1	< 1	< 1	< 1	< 1	< 1	< 1	< 1	< 1	< 1	< 1	< 1	< 1	< 1	**< 1**
16:1	2.3 ±2.6	4.3 ±1.8	3.7 ±1.5	1.8 ±0.6	5.0 ±1.1	4.8 ±1.0	2.3 ±0.9	< 1	4.9 ±0.6	6.8 ±0.6	3.3 ±1.1	1.3 ±1.6	4.8 ±2.2	5.3 ±1.0	1.7 ±0.8	6.9 ±0.4	9.4 ±0.6	**4.1 ±2.3**

16:0	< 1	1.3 ±0.3	< 1	1.5 ±0.2	1.7 ±0.4	1.6 ±0.2	3.0 ±0.8	< 1	2.0 ±0.4	1.1 ±0.2	3.2 ±0.2	< 1	1.3 ±0.3	1.5 ±0.1	2.6 ±0.5	< 1	3.3 ±0.3	**1.7 ±0.9**
i17:1	35.1 ±13.3	43.5 ±8.9	36.4 ±6.8	37.3 ±5.7	46.8 ±5.5	42.3 ±2.7	16.2 ±13.2	17.9 ±4.5	41.4 ±2.6	45.7 ±4.4	27.6 ±3.2	23.2 ±17.9	40.4 ±3.5	42.7 ±1.9	19.2 ±1.4	26.1 ±1.1	19.7 ±0.5	**33.0 ±10.8**
i17:0	2.7 ±0.7	3.2 ±0.7	2.0 ±0.3	3.0 ±0.9	3.1 ±0.4	3.0 ±0.4	18.5 ±4.9	2.9 ±1.3	2.9 ±0.5	2.9 ±0.7	10.5 ±9.1	3.2 ±1.8	3.7 ±0.7	3.7 ±0.4	17.4 ±2.0	1.6 ±0.1	3.1 ±0.3	**5.1 ±5.2**
17:1	6.3 ±7.1	4.8 ±3.3	8.3 ±4.9	5.0 ±1.6	6.3 ±0.9	6.8 ±0.7	4.3 ±2.9	6.4 ±5.6	3.4 ±1.1	1.1 ±0.2	< 1	3.0 ±4.9	3.7 ±4.5	< 1	2.8 ±1.1	3.7 ±0.4	3.7 ±0.9	**4.1 ±2.3**
17:0	5.2 ±0.7	3.9 ±2.7	4.2 ±2.7	6.1 ±1.7	6.6 ±1.7	5.0 ±1.5	3.7 ±1.6	3.5 ±2.7	4.8 ±1.9	1.0 ±0.2	2.2 ±2.3	< 1	< 1	1.1 ±0.2	2.2 ±1.3	1.7 ±0.5	2.1 ±0.2	**3.2 ±1.9**
18:1	5.1 ±4.8	2.6 ±0.5	2.5 ±0.2	4.2 ±0.6	2.3 ±0.2	2.9 ±0.7	2.0 ±0.8	2.4 ±1.0	2.0 ±0.4	1.9 ±0.6	1.5 ±0.2	2.7 ±1.2	1.6 ±0.2	2.1 ±0.4	2.1 ±0.5	< 1	1.5 ±0.1	**2.4 ±1.0**
18:0	3.4 ±1.0	2.7 ±0.5	2.5 ±0.7	4.4 ±0.4	2.9 ±0.3	3.5 ±0.9	2.8 ±0.9	5.2 ±1.5	2.8 ±1.1	1.8 ±0.6	2.7 ±1.1	4.1 ±0.1	1.9 ±0.6	2.5 ±0.5	3.6 ±1.3	< 1	< 1	**2.8 ±1.2**
19:1	< 1	< 1	< 1	1.4 ±0.5	< 1	< 1	< 1	1.6 ±0.4	< 1	< 1	< 1	1.6 ±0.7	< 1	< 1	< 1	< 1	< 1	**< 1**
20:0	< 1	< 1	< 1	1.1 ±0.1	< 1	< 1	< 1	1.8 ±0.6	< 1	< 1	< 1	1.9 ±0.5	< 1	< 1	< 1	< 1	< 1	**< 1**
iDA	n.d.	n.d.	n.d.	n.d.	n.d.	n.d.	n.d.	n.d.	n.d.	n.d.	n.d.	n.d.	n.d.	n.d.	n.d.	n.d.	n.d.	**n.d.**
**Mono/diether relative abundances in % (mean ± 1 standard deviation)**																		
1-i15:0 MAGE	6.4 ±2.5	5.9 ±2.3	11.4 ±5.6	7.9 ±2.9	4.3 ±1.4	6.0 ±0.6	12.3 ±6.1	23.1 ±14.6	1.7 ±0.2	5.5 ±3.5	11.2 ±3.8	21.5 ±14.9	5.2 ±2.7	6.4 ±1.2	11.8 ±4.1	1.8 ±0.1	3.3 ±1.0	**8.6 ±6.1**
2-i15:0 MAGE	2.7 ±1.4	1.4 ±0.3	1.2 ±0.4	2.3 ±1.2	1.5 ±0.2	< 1	< 1	1.9 ±0.5	< 1	< 1	< 1	1.5 ±0.7	< 1	< 1	< 1	< 1	< 1	**1.1 ±0.7**
1,2-i15:0 DAGE	16.6 ±7.3	15.3 ±4.8	15.5 ±4.5	18.9 ±1.0	11.7 ±3.9	15.8 ±3.8	7.4 ±3.7	20.7 ±8.9	9.1 ±2.8	9.7 ±3.4	13.1 ±2.5	27.3 ±10.1	12.6 ±6.9	11.6 ±1.9	11.1 ±9.8	3.7 ±0.7	1.2 ±0.2	**13.0 ±6.2**

Table S4. Liquid chromatography data including all branched GTGTs and branched GDGTs for *S. usitatus* for all experimental conditions. Relative abundances for each sample were calculated from TIC peak areas (n.q. = not quantified due to exceedingly low abundance or complete absence). Reported relative abundances are the statistical means and standard deviations of biological triplicates vs all listed compounds. Note that Fig. 3 visualizes abundances relative to the standard brGDGTs (Table S5) rather than the whole dataset listed here. The last column ('All') represents the statistical average and standard deviation across all experiments. See Fig. 1, Fig. 3 and Table S7 for chemical structures. See Dataset S1 for these data in spreadsheet format.

**Experimental Conditions**																	
**T (°C)**	**15**	**15**	**15**	**20**	**20**	**20**	**20**	**25**	**25**	**25**	**25**	**30**	**30**	**30**	**30**	**25**	**25**	**All**
**pH**	**5.0**	**5.5**	**6.0**	**5.0**	**5.5**	**6.0**	**6.5**	**5.0**	**5.5**	**6.0**	**6.5**	**5.0**	**5.5**	**6.0**	**6.5**	**5.5**	**5.5**	
**% O2**	**21**	**21**	**21**	**21**	**21**	**21**	**21**	**21**	**21**	**21**	**21**	**21**	**21**	**21**	**21**	**5**	**1**	
**Branched GTGT relative abundances in % (mean ± 1 standard deviation)**																		
brGTGT Ia	5.2 ±1.3	4.9 ±0.6	3.4 ±0.6	3.1 ±0.7	5.4 ±1.3	4.0 ±0.6	7.4 ±0.3	2.3 ±1.7	3.8 ±1.4	3.2 ±0.1	9.9 ±3.6	1.8 ±0.7	1.7 ±0.1	1.3 ±0.1	6.2 ±1.9	2.0 ±0.6	1.6 ±0.3	**4.0 ±2.3**
brGTGT IIa	0.31 ±0.16	0.18 ±0.05	0.12 ±0.04	1.1 ±0.4	0.53 ±0.32	0.21 ±0.03	0.09 ±0.02	0.99 ±1.53	1.7 ±0.1	0.35 ±0.02	0.11 ±0.04	0.14 ±0.13	0.28 ±0.01	0.18 ±0.02	0.06 ±0.01	0.69 ±0.11	0.44 ±0.16	**0.44 ±0.45**
brGTGT IIIa	0.20 ±0.08	0.14 ±0.04	0.11 ±0.03	0.49 ±0.14	0.41 ±0.10	0.17 ±0.01	0.11 ±0.01	0.53 ±0.56	1.1 ±0.5	0.28 ±0.01	0.11 ±0.04	0.32 ±0.04	0.33 ±0.01	0.22 ±0.03	0.19 ±0.01	3.8 ±0.9	0.91 ±0.22	**0.56 ±0.88**
**Standard branched GDGT relative abundances in % (mean ± 1 standard deviation)**																		
brGDGT Ia	55 ±2	51 ±3	42 ±2	80 ±3	76 ±2	74 ±0	72 ±0	92 ±3	90 ±2	93 ±0	89 ±4	95 ±1	96 ±0	96 ±0	93 ±2	82 ±1	89 ±1	**80 ±17**
brGDGT Ib	0.40 ±0.05	0.51 ±0.09	0.40 ±0.15	1.2 ±0.5	0.90 ±0.33	1.0 ±0.2	0.26 ±0.03	1.9 ±1.1	1.2 ±0.2	1.9 ±0.1	0.22 ±0.02	1.9 ±0.2	1.9 ±0.1	1.6 ±0.1	0.18 ±0.00	1.2 ±0.1	0.33 ±0.07	**1.0 ±0.7**
brGDGT Ic	n.q.	n.q.	n.q.	n.q.	n.q.	n.q.	n.q.	n.q.	n.q.	n.q.	n.q.	n.q.	n.q.	n.q.	n.q.	n.q.	n.q.	**n.q.**

brGDGT IIa	36 ±1	40 ±2	48 ±2	13 ±2	16 ±1	20 ±1	18 ±0	1.7 ±0.3	1.6 ±0.0	1.3 ±0.1	1.0 ±0.1	0.58 ±0.39	0.15 ±0.01	0.21 ±0.00	0.19 ±0.01	5.4 ±0.1	2.5 ±0.2	**12 ±16**
brGDGT IIb	0.25 ±0.04	0.44 ±0.16	0.43 ±0.24	n.q.	0.16 ±0.07	0.23 ±0.03	0.04 ±0.00	n.q.	n.q.	n.q.	n.q.	n.q.	n.q.	n.q.	n.q.	n.q.	n.q.	**0.09 ±0.16**
brGDGT IIc	n.q.	n.q.	n.q.	n.q.	n.q.	n.q.	n.q.	n.q.	n.q.	n.q.	n.q.	n.q.	n.q.	n.q.	n.q.	n.q.	n.q.	**n.q.**
brGDGT IIIa	2.9 ±0.2	3.3 ±0.4	5.2 ±0.6	0.99 ±1.24	0.30 ±0.02	0.46 ±0.04	2.4 ±0.1	0.04 ±0.01	0.05 ±0.03	0.05 ±0.01	0.05 ±0.01	0.07 ±0.05	0.03 ±0.00	0.03 ±0.00	0.06 ±0.00	0.03 ±0.00	0.01 ±0.00	**0.94 ±1.55**
brGDGT IIIb	n.q.	n.q.	n.q.	n.q.	n.q.	n.q.	n.q.	n.q.	n.q.	n.q.	n.q.	n.q.	n.q.	n.q.	n.q.	n.q.	n.q.	**n.q.**
brGDGT IIIc	n.q.	n.q.	n.q.	n.q.	n.q.	n.q.	n.q.	n.q.	n.q.	n.q.	n.q.	n.q.	n.q.	n.q.	n.q.	n.q.	n.q.	**n.q.**
**Uncommon branched GDGT relative abundances in % (mean ± 1 standard deviation)**																		
brGDGT IIIa-2	n.q.	n.q.	n.q.	0.05 ±0.01	0.03 ±0.01	0.02 ±0.00	n.q.	0.04 ±0.02	0.07 ±0.03	0.02 ±0.00	0.01 ±0.00	0.03 ±0.00	0.03 ±0.00	0.03 ±0.00	0.04 ±0.00	2.3 ±0.2	3.5 ±0.4	**0.36 ±0.97**
brGDGT IIIb-2	n.q.	n.q.	n.q.	0.14 ±0.02	0.08 ±0.04	0.02 ±0.00	0.01 ±0.00	0.14 ±0.15	0.26 ±0.10	0.02 ±0.00	0.01 ±0.00	0.05 ±0.01	0.04 ±0.00	0.04 ±0.00	0.07 ±0.01	2.7 ±0.2	1.7 ±0.2	**0.31 ±0.73**

Table S5. Calculated methylation index of brGDGTs (MBT'_{5Me}); cyclization index of brGDGTs (CBT_{5Me}); degree of cyclization (DC); and branched GDGT abundances relative to standard branched GDGTs (% of brGDGTs, abbreviated as %br in main text). Because the degree of cyclization is very low for all samples, DC is listed here in % (i.e. x 100). See Dataset S1 for all data in spreadsheet format.

$$MBT'_{5Me} = (Ia + Ib + Ic) / (Ia + Ib + Ic + IIa + IIb + IIc + IIIa)$$

$$CBT_{5Me} = - \log((Ib + IIb) / (Ia + IIa))$$

$$DC\ [\%] = (Ib + 2 * Ic + IIb + IIb') / (Ia + Ib + Ic + IIa + IIa' + IIb + IIb') * 100$$

$$\%br = [(brGDGTx) / (Ia + Ib + Ic + IIa + IIb + IIc + IIIa + IIIb + IIIc)] * 100$$

T (°C)	15	15	15	20	20	20	20	25	25	25	25	30	30	30	30	25	25
pH	5.0	5.5	6.0	5.0	5.5	6.0	6.5	5.0	5.5	6.0	6.5	5.0	5.5	6.0	6.5	5.5	5.5
% O2	21	21	21	21	21	21	21	21	21	21	21	21	21	21	21	5	1
**Indices**																	
MBT'5Me	0.58 ±0.01	0.54 ±0.03	0.44 ±0.02	0.85 ±0.02	0.82 ±0.02	0.79 ±0.01	0.78 ±0.00	0.98 ±0.00	0.98 ±0.00	0.99 ±0.00	0.99 ±0.00	0.99 ±0.00	1.0 ±0.0	1.0 ±0.0	1.0 ±0.0	0.94 ±0.00	0.97 ±0.00
CBT5Me	2.1 ±0.1	2.0 ±0.1	2.1 ±0.2	1.9 ±0.2	2.0 ±0.2	1.9 ±0.1	2.5 ±0.0	1.7 ±0.2	1.9 ±0.1	1.7 ±0.0	2.6 ±0.0	1.7 ±0.0	1.7 ±0.0	1.8 ±0.0	2.7 ±0.0	1.9 ±0.0	2.4 ±0.1
DC [%]	0.71 ±0.10	1.0 ±0.3	0.92 ±0.43	1.2 ±0.5	1.1 ±0.4	1.3 ±0.2	0.33 ±0.03	2.0 ±1.0	1.3 ±0.2	2.0 ±0.1	0.24 ±0.02	1.9 ±0.2	2.0 ±0.1	1.7 ±0.1	0.20 ±0.00	1.3 ±0.1	0.36 ±0.09
**Branched GDGT abundances relative to standard branched GDGTs (%br)**																	
brGDGT Ia	57.8 ±1.3	53.5 ±3.3	43.9 ±1.9	83.7 ±2.1	81.1 ±2.0	77.6 ±0.7	78.1 ±0.4	96.3 ±1.3	97.0 ±0.1	96.6 ±0.1	98.6 ±0.1	97.4 ±0.2	97.9 ±0.1	98.1 ±0.1	99.5 ±0.0	92.5 ±0.1	96.9 ±0.3
brGDGT Ib	0.43 ±0.05	0.54 ±0.10	0.42 ±0.16	1.22 ±0.50	0.97 ±0.35	1.07 ±0.18	0.28 ±0.03	1.96 ±1.04	1.31 ±0.17	2.00 ±0.10	0.24 ±0.02	1.92 ±0.20	1.96 ±0.10	1.67 ±0.11	0.20 ±0.00	1.31 ±0.12	0.36 ±0.09
brGDGT Ic	n.q.	n.q.	n.q.	n.q.	n.q.	n.q.	n.q.	n.q.	n.q.	n.q.	n.q.	n.q.	n.q.	n.q.	n.q.	n.q.	n.q.
brGDGT IIa	38.4 ±1.0	42.1 ±2.6	49.9 ±1.6	14.0 ±1.7	17.5 ±1.6	20.7 ±0.6	19.0 ±0.2	1.71 ±0.28	1.68 ±0.02	1.37 ±0.07	1.14 ±0.10	0.59 ±0.40	0.16 ±0.01	0.21 ±0.00	0.21 ±0.01	6.15 ±0.18	2.77 ±0.26
brGDGT IIb	0.26 ±0.04	0.47 ±0.17	0.45 ±0.25	n.q.	0.17 ±0.08	0.25 ±0.03	0.04 ±0.00	n.q.	n.q.	n.q.	n.q.	n.q.	n.q.	n.q.	n.q.	n.q.	n.q.

brGDGT IIc	n.q.	n.q.	n.q.	n.q.	n.q.	n.q.	n.q.	n.q.	n.q.	n.q.	n.q.	n.q.	n.q.	n.q.	n.q.	n.q.	n.q.
brGDGT IIIa	3.11 ±0.26	3.44 ±0.48	5.39 ±0.65	1.04 ±1.31	0.32 ±0.02	0.48 ±0.04	2.60 ±0.15	0.04 ±0.01	0.05 ±0.03	0.05 ±0.01	0.05 ±0.01	0.08 ±0.05	0.03 ±0.00	0.04 ±0.00	0.06 ±0.00	0.03 ±0.00	0.01 ±0.00
brGDGT IIIb	n.q.	n.q.	n.q.	n.q.	n.q.	n.q.	n.q.	n.q.	n.q.	n.q.	n.q.	n.q.	n.q.	n.q.	n.q.	n.q.	n.q.
brGDGT IIIc	n.q.	n.q.	n.q.	n.q.	n.q.	n.q.	n.q.	n.q.	n.q.	n.q.	n.q.	n.q.	n.q.	n.q.	n.q.	n.q.	n.q.

Table S6. Protein BLAST results from the *S. usitatus* Ellin6076 proteome (https://www.uniprot.org/proteomes/UP000000671, retrieved Feb. 27 2022) for proteins potentially involved in brGDGT biosynthesis (e-value < $1e^{-10}$). See Figure S12 for domain visualizations.

Protein	Query				BLASTP results from *S. usitatus* Ellin6076					
	**Organism**	**Gene Locus**	**UniProt ID**	**AAs**	**Gene Locus**	**UniProt ID**	**AAs**	**Coverage**	**Identity**	**e-value**
PlsA	*Clostridium perfringens*	CPE1195	Q8XL47	1004	Acid_0922	Q02AJ5	1188	96%	30.2%	1.48e-126
PlsR	*Clostridium perfringens*	CPE1194	Q8XL48	420	Acid_0921	Q02AJ6	589	96%	20.9%	2.33e-14
Tes	*Methanosarcina acetivorans*	MA_1486	Q8TQQ4	509	Acid_5929	Q01U00	545	96%	40.2%	1.38e-139
					Acid_2410	Q025C7	714	92%	30.1%	4.18e-62
GrsA	*Sulfolobus acidocaldarius*	Saci_1585	Q4J8I0	489	Acid_5783	Q01UE0	597	75%	29.3%	3.90e-38
GrsB	*Sulfolobus acidocaldarius*	Saci_0240	Q4JC22	528	Acid_5783	Q01UE0	597	84%	25.2%	1.29e-40
ElbD	*Myxococcus xanthus*	MXAN_1528	Q1DC43	1470	Acid_7444	Q01PR8	597	37%	26.4%	1.29e-57
					Acid_0997	Q02AC7	468	31%	30.9%	4.88e-51
					Acid_5700	Q01UM3	554	30%	30.0%	1.80e-45
					Acid_3608	Q020R4	540	36%	27.7%	6.05e-44
					Acid_1327	Q029G6	496	32%	27.7%	1.55e-04

Table S7. Chemical structures of compounds discussed in the manuscript.

FAs (fatty acids)

i15:0 FA (*iso* C15:0 fatty acid)

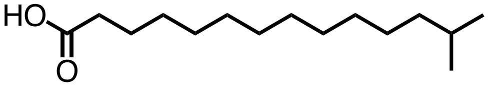

iDA (*iso* diabolic acid / 13,16-dimethyl-octacosanedioic acid)

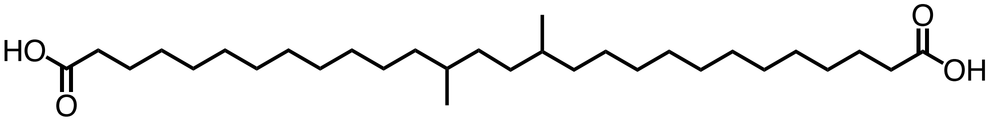

MAGEs (monoalkyl/monoalkanoic glycerol monoethers)

i15:0 MAGE (1-*iso* C15:0 monoalkyl glycerol monoether) aka 1-*iso* C15 MGE

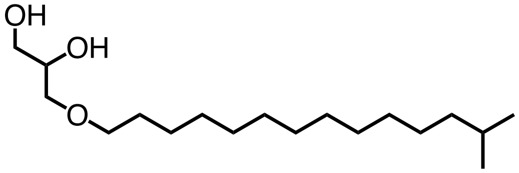

i15:0 MAGE (2-*iso* C15:0 monoalkyl glycerol monoether) aka 2-*iso* C15 MGE

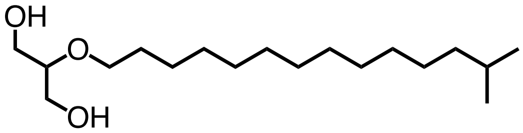

iDA MAGE (*iso*-diabolic acid monoalkanoic glycerol monoether) aka *iso*-diabolic acid MGE

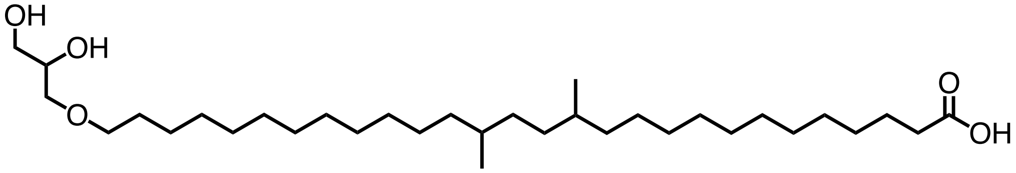

DAGEs (dialkyl/dialkanoic glycerol diethers)

1,2-i15:0 DAGE (1,2-*iso* C15:0 dialkyl glycerol diether) aka 1,2-*iso* C15 DGE

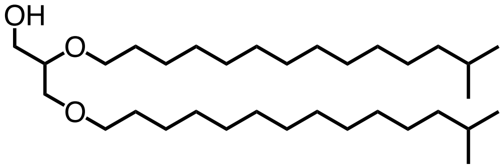

1,2-iDA DAGE (1,2-*iso* diabolic acid dialkanoic glycerol diether)

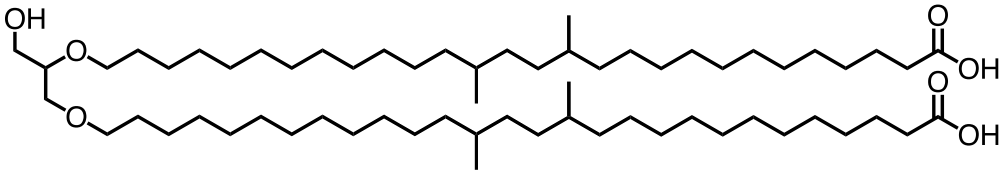

GTGTs (glycerol trialkyl glycerol tetraethers)

brGTGT Ia

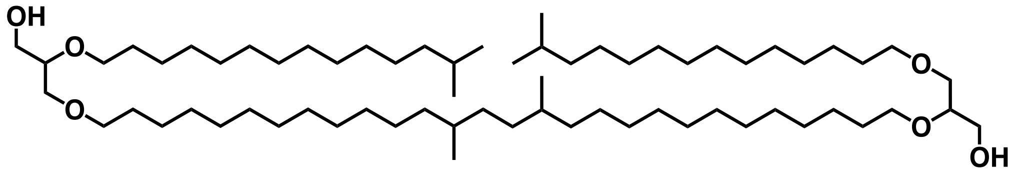

brGTGT IIa

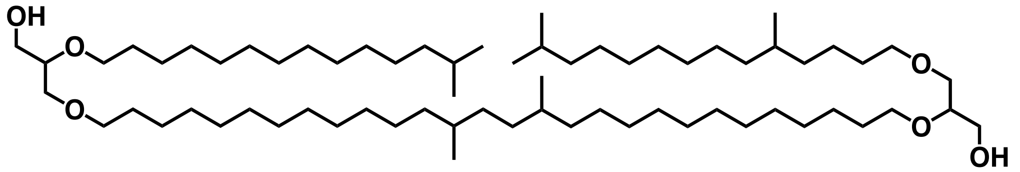

brGTGT IIIa-2 (proposed potential structures)

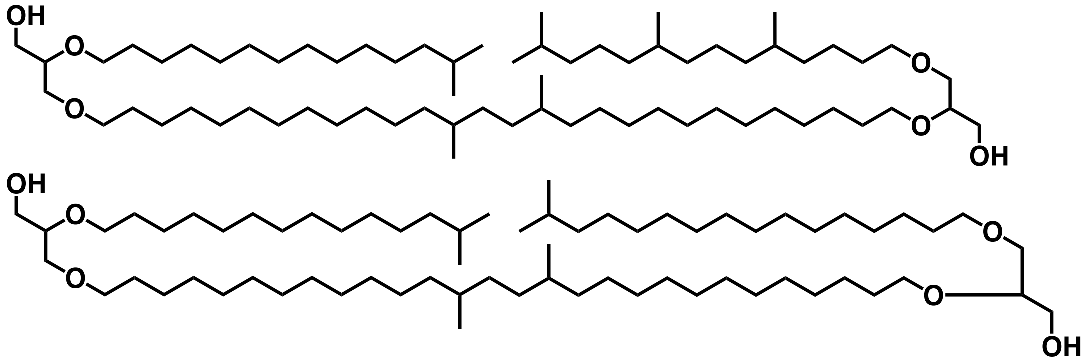

GDGTs (glycerol dialkyl glycerol tetraethers)

brGDGT Ia

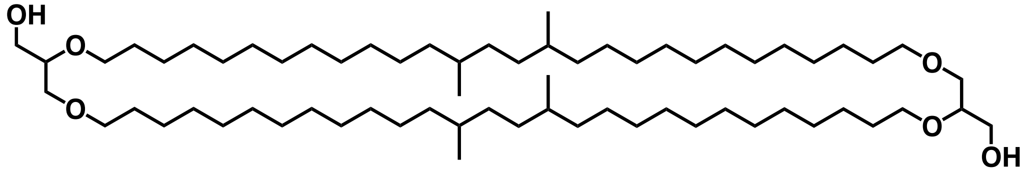

brGDGT Ib

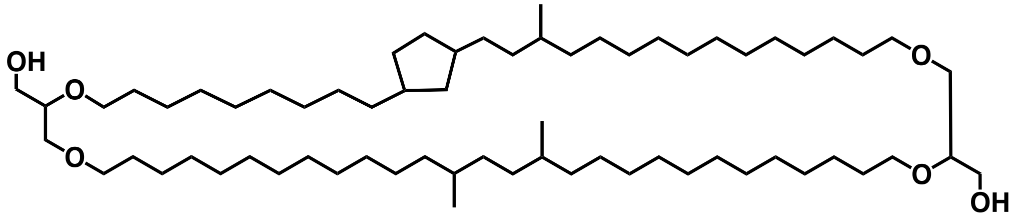

brGDGT Ic

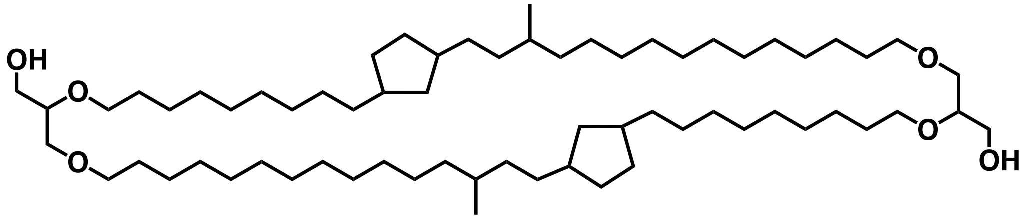

brGDGT IIa

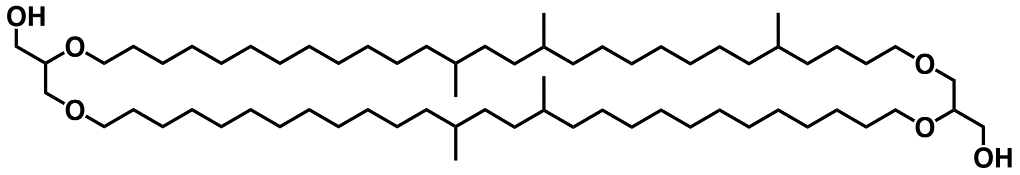

brGDGT IIb

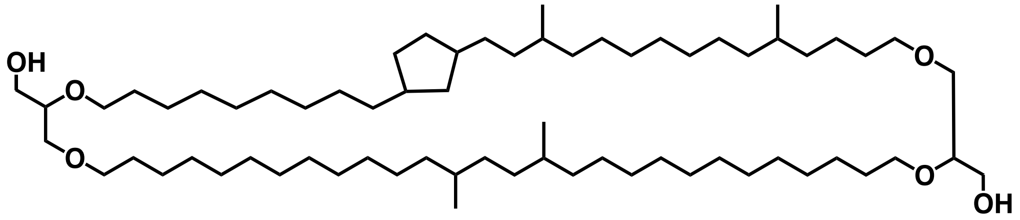

brGDGT IIc

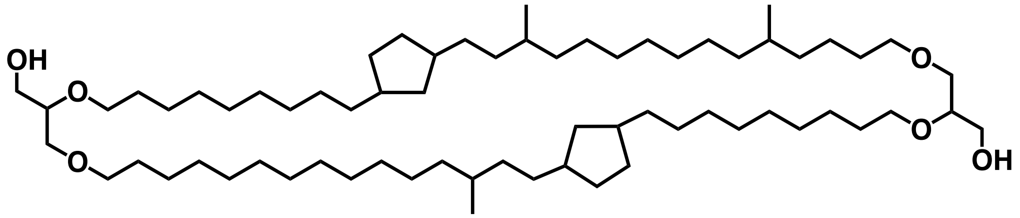

brGDGT IIIa

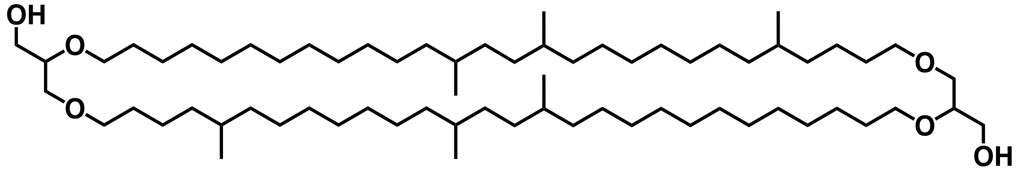

brGDGT IIIa' / $IIIa_6$

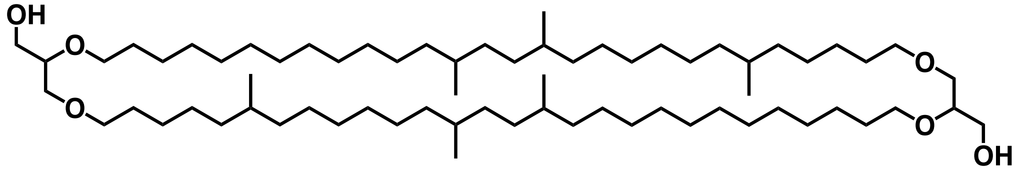

brGDGT $IIIa_7$

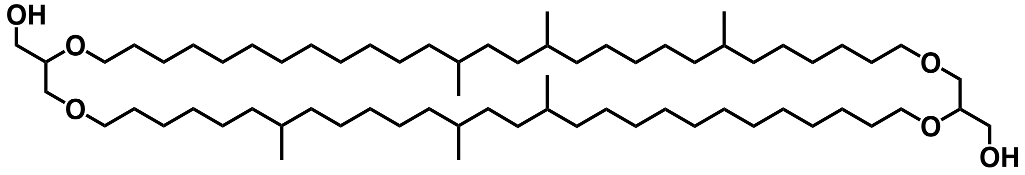

brGDGT IIIb

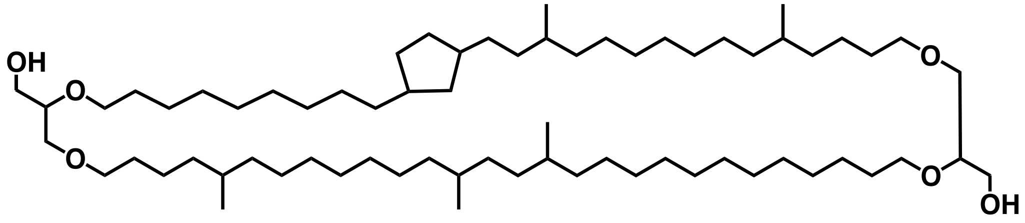

brGDGT IIIb' / $IIIb_6$

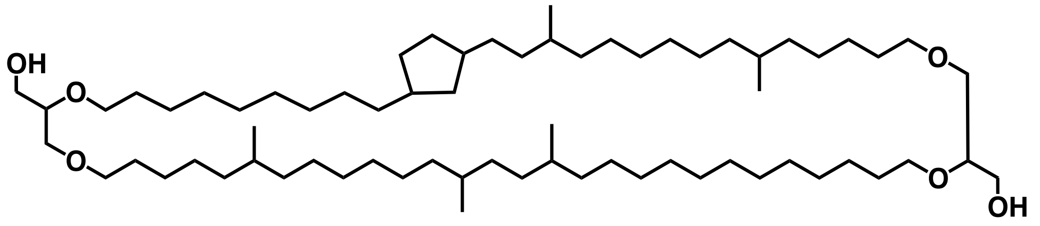

brGDGT IIIb₇

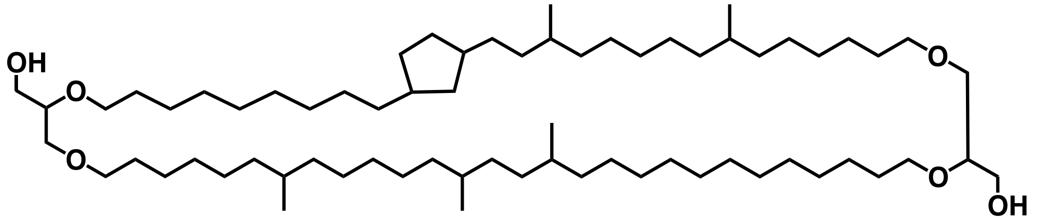

brGDGT IIIc

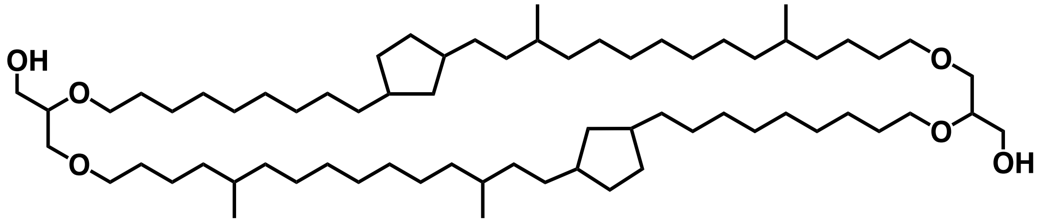

Uncommon GDGTs

brGDGT IIIa-2 (proposed potential structures)

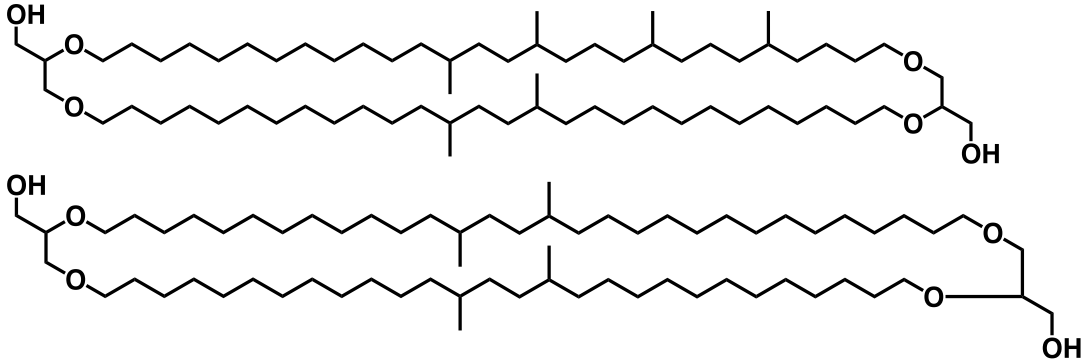

brGDGT IIIb-2 (proposed potential structures)

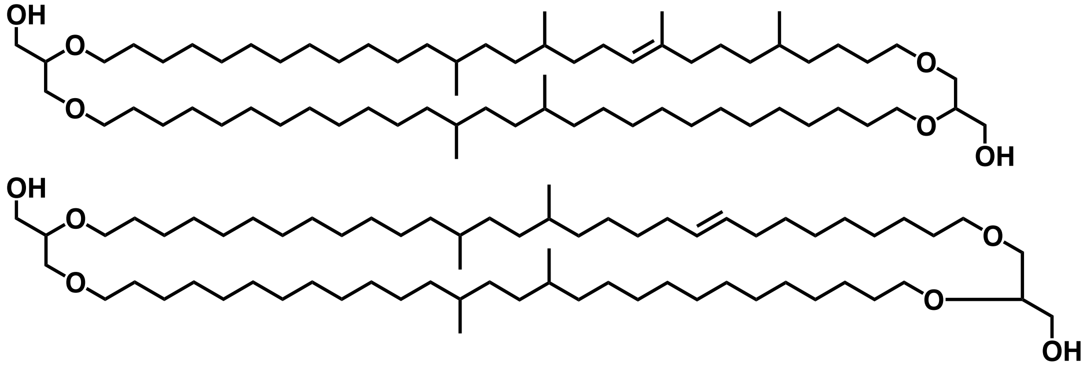

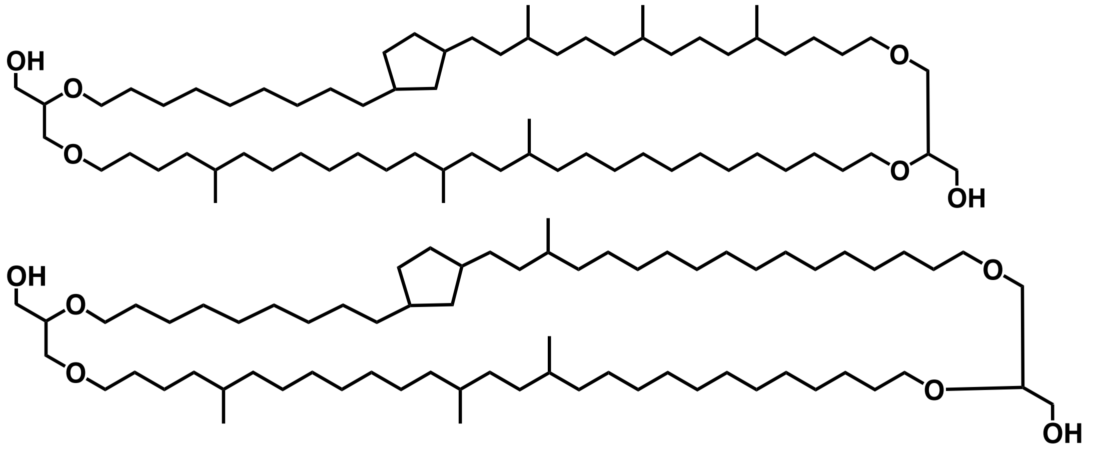
OH
O
O
O
O
OH
OH
O
O
O
O
OH

SI References

Baxter, A. J., Hopmans, E. C., Russell, J. M., & Sinninghe Damsté, J. S. (2019). Bacterial GMGTs in East African lake sediments: Their potential as palaeotemperature indicators. *Geochimica et Cosmochimica Acta*, *259*, 155–169. https://doi.org/10.1016/j.gca.2019.05.039

Hunter, S., Apweiler, R., Attwood, T. K., Bairoch, A., Bateman, A., Binns, D., Bork, P., Das, U., Daugherty, L., Duquenne, L., Finn, R. D., Gough, J., Haft, D., Hulo, N., Kahn, D., Kelly, E., Laugraud, A., Letunic, I., Lonsdale, D., … Yeats, C. (2009). InterPro: The integrative protein signature database. *Nucleic Acids Research*, *37*(Database), D211–D215. https://doi.org/10.1093/nar/gkn785

Mi, H., Muruganujan, A., & Thomas, P. D. (2012). PANTHER in 2013: Modeling the evolution of gene function, and other gene attributes, in the context of phylogenetic trees. *Nucleic Acids Research*, *41*(D1), D377–D386. https://doi.org/10.1093/nar/gks1118

Mistry, J., Chuguransky, S., Williams, L., Qureshi, M., Salazar, G. A., Sonnhammer, E. L. L., Tosatto, S. C. E., Paladin, L., Raj, S., Richardson, L. J., Finn, R. D., & Bateman, A. (2021). Pfam: The protein families database in 2021. *Nucleic Acids Research*, *49*(D1), D412–D419. https://doi.org/10.1093/nar/gkaa913

Raberg J. H., Miller G. H., Geirsdóttir Á. and Sepúlveda J. (2022a) [in press], Near-universal trends in brGDGT lipid distributions in nature.

Raberg, J. H., Harning, D. J., Crump, S. E., de Wet, G., Blumm, A., Kopf, S., Geirsdóttir, Á., Miller, G. H., & Sepúlveda, J. (2021). Revised fractional abundances and warm-season temperatures substantially improve brGDGT calibrations in lake sediments. *Biogeosciences*, *18*(12), 3579–3603. https://doi.org/10.5194/bg-18-3579-2021
